# Upon heat stress processing of ribosomal RNA precursors into mature rRNAs is compromised after cleavage at primary P site in *Arabidopsis thalian*a

**DOI:** 10.1080/15476286.2022.2071517

**Published:** 2022-05-06

**Authors:** T. Darriere, E. Jobet, D. Zavala, M.L. Escande, N. Durut, A. de Bures, F. Blanco-Herrera, E.A. Vidal, M. Rompais, C. Carapito, S. Gourbiere, J. Sáez-Vásquez

**Affiliations:** a CNRS, Laboratoire Génome et D#x0E9;veloppement des Plantes (LGDP), UMR 5096, 66860 Perpignan, France; b Univ. Perpignan Via Domitia, LGDP, UMR 5096, Perpignan, France; c Centro de Biotecnología Vegetal, Facultad de Ciencias de la Vida, Universidad Andres Bello, Santiago, Chile; d CNRS, Observatoire Océanologique de Banyuls s/ mer, Banyuls-sur-mer, France; e BioPIC Platform of the OOB, Banyuls-sur-mer, France; f Millennium Institute for Integrative Biology (IBio), Santiago, Chile; g Bioinformática, Facultad de Ciencias, Universidad MayorCentro de Genómica y , Santiago, Chile; h Laboratoire de Spectrométrie de Masse BioOrganique, Institut Pluridisciplinaire Hubert Curien, UMR7178 CNRS/Université de Strasbourg, Strasbourg, France

**Keywords:** Nucleolus, heat stress, rRNA processing, ribosome, Arabidopsis

## Abstract

Transcription and processing of 45S rRNAs in the nucleolus are keystones of ribosome biogenesis. While these processes are severely impacted by stress conditions in multiple species, primarily upon heat exposure, we lack information about the molecular mechanisms allowing sessile organisms without a temperature-control system, like plants, to cope with such circumstances. We show that heat stress disturbs nucleolar structure, inhibits pre-rRNA processing and provokes imbalanced ribosome profiles in *Arabidopsis thaliana* plants. Notably, the accuracy of transcription initiation and cleavage at the primary P site in the 5’ETS (5’ External Transcribed Spacer) are not affected but the levels of primary 45S and 35S transcripts are, respectively, increased and reduced. In contrast, precursors of 18S, 5.8S and 25S RNAs are rapidly undetectable upon heat stress. Remarkably, nucleolar structure, pre-rRNAs from major ITS1 processing pathway and ribosome profiles are restored after returning to optimal conditions, shedding light on the extreme plasticity of nucleolar functions in plant cells. Further genetic and molecular analysis to identify molecular clues implicated in these nucleolar responses indicate that cleavage rate at P site and nucleolin protein expression can act as a checkpoint control towards a productive pre-rRNA processing pathway.

## Introduction

Ribosomal RNAs (rRNAs) are the ribosomes’ structural and functional building blocks. rRNA gene units (rDNA) 35S in yeast, 45S in plants, and 47S in mammals, encode the 18S, 5.8S and 25S (28S in mammals) rRNAs. Each rDNA contains external transcribed (5’ETS and 3’ETS) and the 18S, 5.8S and 25/28S rRNAs separated by internal transcribed spacer (ITS1 and ITS2). Each rDNA unit is transcribed by RNA polymerase I (Pol I) in the nucleolus as a single 35S/45S/47S pre-rRNA processed into mature 18S, 5.8S, and 25S/28S rRNAs. Processing of pre-rRNAs consists of exo- and endonucleolytic cleavages to remove ETS and ITS sequences and RNA modifications at specific positions [1–4].

In *Arabidopsis thaliana*, the initial endonucleolytic cleavage of the 45S pre-rRNA is located at the P site in the 5’ETS [5]. This cleavage is the equivalent of yeast A_0_ and mammalian 01(A’) [1,6] and generates the 35S pre-rRNA (reviewed in [4,7]). The endonucleases that cleave at 5’ETS have yet to be assigned, but cleavage at the A_0_/A0 (in yeast and mammals) and 01/A′ (in mammals) sites require the U3 snoRNP complex [8]. In cruciferous plants, a nucleolin-U3 snoRNP protein complex produces an accurate cleavage at the P (the equivalent of 01/A′) site *in vitro* [5]. The 5’ETS from Arabidopsis does not include any sequence with significant complementarity to the U3 hinge region that might be required for cleavage at the P site, as demonstrated in yeast [9]. However, nucleolin gene disruption induces accumulation of pre-rRNA cleaved at the P site in Arabidopsis [10]. Due to a 1.2 kb insertion, the Arabidopsis 45S pre-rRNA has a much longer 5’ETS than pre-rRNAs from yeast and mammalian cells, or even from other plants. The exonuclease XRN2 then shortens it prior to cleavage at the P site [11]. The Arabidopsis 35S is easily detected [11–14], suggesting that cleavages at P, P’ in the 5’ETS and A_3_ in the ITS1 occur post-transcriptionally, as in mammalian cells (post-transcriptional cleavages at sites 01(A’), A0, and site 2), but in contrast to yeast (co-transcriptional cleavages at A_0_, A_1_, and A_2_) [1,15].

Two alternative 35S pre-rRNA processing pathways co-exist in *A. thaliana*. In the major ITS1-first pathway, the 35S is first cleaved at the A3 site and then at the P’ and P2 sites, while in the minor 5’ETS-first pathway, the 35S intermediate is first cleaved at the P’ and P2 sites, and then at the A2 site [11,16]. Two similar alternative pre-rRNA processing pathways also occur in mammals [1]. In yeast, only co-transcriptional cleavage at A_2_ in the ITS1 is productive (A_2_ pathway) since cleavage at A_3_ results in the production of pre-rRNAs degraded by TRAMP/Exosome (non-productive A_3_ pathway) [17]. Then, the Arabidopsis major ITS1 pathway is analogous to the main processing pathway in mammalian cells, while the minor 5’ETS pathway is comparable to the productive A_2_ pathway in yeast. Specific pre-RNA transcripts were detected in the Arabidopsis *irp7* mutants upon auxin treatment and in fast-dividing cell cultures, proposing alternative pre-rRNA processing pathways in plants [18].

Environmental and cell stress conditions induce known changes in nucleolar morphology and functions [19–22]. However, the impact of heat stress on the processing of pre-rRNAs remains poorly investigated. In mammals, a short heat shock inhibits pre-rRNA transcription and processing into mature rRNAs [23], while 40 min exposure at 43°C causes accumulation of 30SL pre-RNAs from the ITS1-first pathway [24]. In Arabidopsis, the accumulation of specific pre-rRNAs from the major ITS1 pathway becomes evident in *rh10* [25] and *rid3* [26] mutant plants only after mild heat stress. Taken together, these observations led us to investigate pre-rRNA processing in *Arabidopsis thaliana* seedlings exposed for several hours to 37°C and then after returning to optimal growing conditions. We thus examined nucleolus structure, characterized rRNA transcripts, and studied the impact on ribosome subunit assembly.

## Results

### Heat stress causes nucleolus disorganization

**Figure 1.**
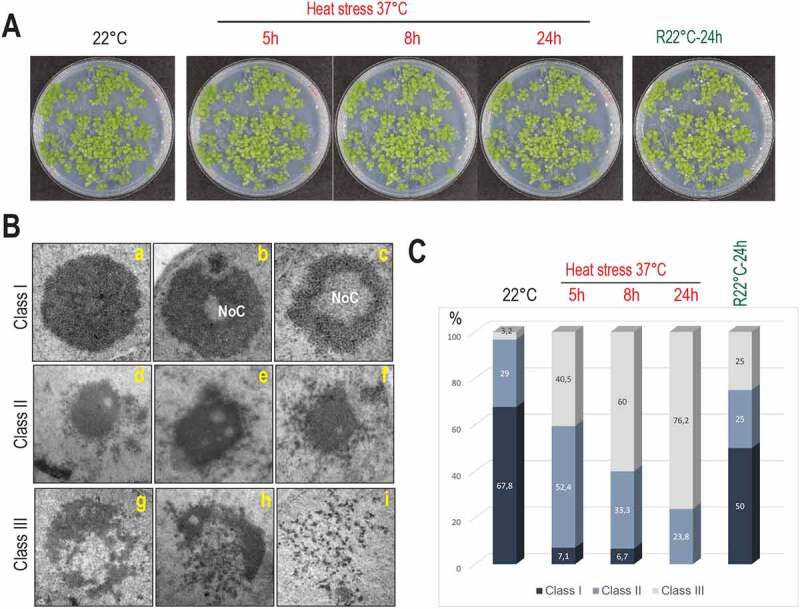
Plant growth and nucleolus organization in response to heat stress. A) Arabidopsis seedlings maintained at 22°C (control), heat treated (37°C for 5 h, 8 h, and 24 h) or recovered R22°C-24 h (24 h at 37°C and then transferred 24 h at 22°C). **B)** Nucleolus structures visualized by TEM at 22°C and 37°C. Class I, regular nucleoli without (A) or with (B and C) Nucleolar Cavities (NoC). Class II, partially disrupted nucleoli (D-F); Class III, open (G and H) and entirely disrupted (I) nucleoli. **C)** Bar graph distribution (%, from 116 total nucleoli) of class I (dark blue), II (light blue), and III (white) nucleoli in non-treated control (22°C), heat treated (37°C), and recovered (R22°C-24 h) seedlings.

The nucleolus is the most prominent nuclear structure, and it has been recognized as a central hub in cellular stress response [21,27]. We thus investigated the impact of prolonged heat stress on plant growth and nucleolar structure. Firstly, we observed that seedlings exposed at 37°C for 2 h to 55 h did not show obvious phenotypic growth changes before 24 h-30 h, indicating that Arabidopsis Col-0 ecotype is able to adapt/resist to the applied high-temperature treatment. The phenotype of the recovered seedlings (after 24 h, 29 h, or 31 h at 22°C) was also similar to seedlings before or after 24 h at 37°C (Fig. 1A and S1).

We then determined nucleolus morphology in non-treated (22°C), heat treated (37°C for 5 h, 8 h and 24 h) and heat treated then recovered seedlings (R22°C-24 h). Based on 116 TEM images (Figure S2), we defined three nucleolus organization classes (Fig. 1B). Class I: round shaped nucleoli without (inset a) or with a large (inset b) or small (inset c) Nucleolar Cavity (NoC) [28]; the class II, an intermediate state with dispersed and much less granulated nucleoli (insets d-f); and the class III, “open’ (insets g and h) or collapsed (inset i) nucleoli. The bar graph (Fig. 1C) shows the distribution (%) of class I, II, and III nucleoli in non-treated, heat treated, and recovered seedlings. Under optimal growth conditions (22°C), 67.8%, 29% and 3.2% of the nucleoli belonged, respectively, to class I, II and III while under heat stress conditions, the percent of nucleoli class I, II, and III were progressively reversed: 7.1%, 52.4% and 40.5% after 5 h, 6.7%, 33.3% and 60% after 8 h and 0%, 23.8% and 76.2% after 24 h at 37°C. Notably, in the recovered seedlings (R22°C, 24 h), the percentage of nucleoli class I, II, and III were, respectively, 50%, 25%, and 25%, approaching % ratios observed before heat treatment (control, 22°C).

Together, these data reveal that heat stress induces a strong nucleolar disorganization, which is re-established after returning to optimal growth conditions and suggest a nucleolar plasticity in response to heat stress.

### Divergent accumulation of primary 45S and pre-35S rRNA in response to heat stress

Transcription and processing of 45S rRNA precursors precede the assembly of a visible nucleolus in plants [28] and animal cells [29]. Therefore, as a prolonged heat stress treatment almost completely disrupted the nucleolus in Arabidopsis seedlings, we examined the expression of specific 45S rRNA genes and the accumulation of primary pre-rRNA precursors transcribed by RNA Pol I from transcription initiation site (TIS) and pre-rRNAs processed at the primary cleavage site (P site) in the 5’ETS.

**Figure 2.**
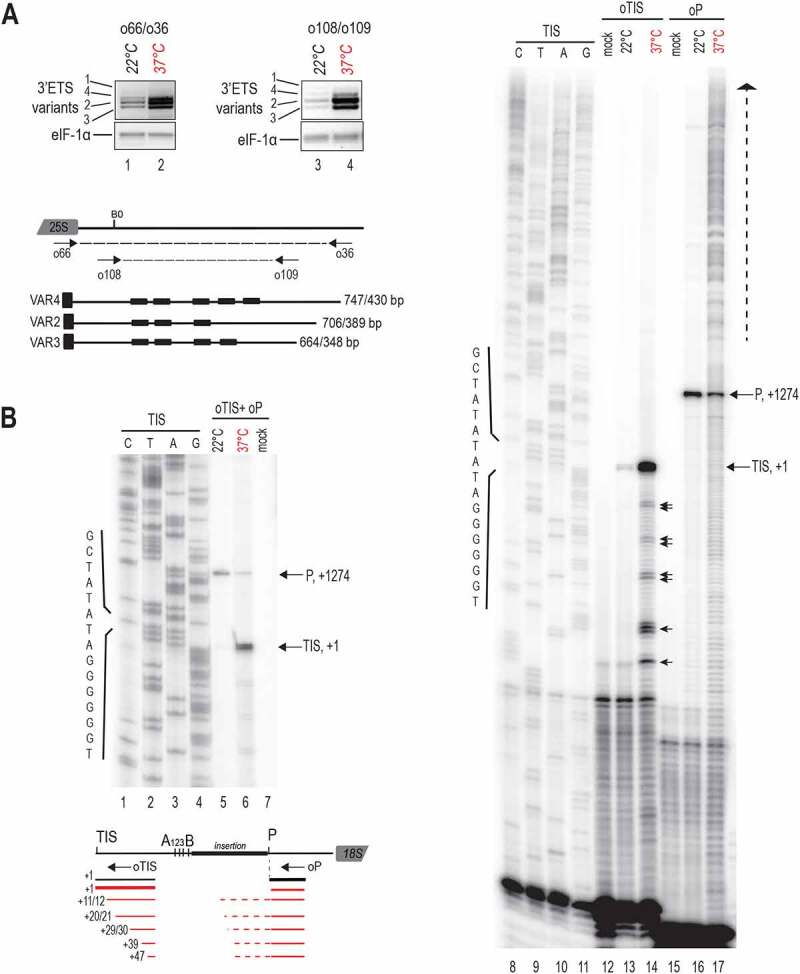
Expression of 3’ETS rDNA variants and analysis of TIS and P site in the 5’ETS of rRNA sequences under heat stress. A) RT-PCR using cDNAs prepared from non-treated (22°C, lanes 1 and 3) and heat treated (24 h, 37°C, lanes 2 and 4) plants. 3’ETS schematic representation below shows R1-4 repeat sequences, primers pairs *o66/o36* and *o108/o109*, and expected amplification sequences. Amplification of eIF1α transcripts was performed to verify similar amounts of cDNA in each sample. B) Primer extension assays were performed on total RNAs from non-treated (22°C) and heat treated (37°C for 24 h) seedlings. Primers oTIS and oP detect respectively Transcription Initiation Site (TIS at +1) and the P cleavage site (at +1274). Reactions with oTIS+oP detects simultaneously TIS and P sites at 22°C and 37°C (lanes 5 and 6). Reactions with oTIS or oP only detects TIS and P sites at 22°C (lanes 13 and 16) or at 37°C (lanes 14 and 17). Lanes 1–4 and 8–11 are rDNA sequencing reactions with primer oTIS used to map transcription from TIS and rRNAs cleaved at P. Lanes 7, 12 and 15, mock control reactions using yeast tRNA. Schemes below show relative positions of primers oTIS and oP and the rRNA species detected at 22°C and 37°C.

In *A. thaliana* Col-0, 45S rDNA units localize in Nucleolus Organizer Regions of chromosomes 2 and 4 (NOR2 and 4). Only rDNA from NOR4 (variants 2–3) is expressed in standard plant growth conditions [30,31] and only rRNAs from NOR4 contribute to the pool of ribosomes even in plants having both NOR2 and NOR4 transcriptionally active [32]. Significantly, rRNA transcribed for NOR2 or 4 can be detected by RT-PCR amplification of 3’ETS sequences. Therefore, to study expression from four distinct 45S rRNA gene variants in Arabidopsis, we performed RT-PCR on total RNAs from control non-treated (22°C) and heat treated (37°C for 24 h) seedlings (Fig. 2A). We used primers o66/o36 to detect 45S pre-rRNA and o108/o109 to detect 45S pre-rRNAs and 3’ETS cleave-off products [33]. At 22°C primer couples o66/o36 (lanes 1 and 2) and o108/o109 (lanes 3 and 4) detected transcription of major 45S rDNA VAR2 and VAR3 and minor rDNA VAR4 from NOR2. Residual rRNA VAR 1 transcripts can be detected as well 22°C (lanes 1 and 3). Upon heat stress, both primer pairs detected the accumulation of VAR2-4 3’ETS transcripts. In contrast, rRNA VAR1 from NOR2 did not accumulate (lanes 2 and 4).

Transcription of 45S rRNA and early cleavages in the 5’ETS are tightly coordinated [5,34]. Therefore, we determined the impact of heat stress on 45S rRNA transcription from initiation site (TIS) at +1 and cleavage at the P site at +1274 in the 5’ETS [10]. We performed primer extension assays on total RNAs from control non-treated (22°C) and heat treated (37°C for 24 h) seedlings with primers oTIS and/or oP (Table S1) to map, respectively, transcription TIS and P sites (Fig. 2B). Primer extension using single primers (oTIS or oP) or both (oTIS+oP) mapped accurately TIS (lanes 5, 6, 13 and 14) and P site (lanes 5, 6, 16 and 17) at 22°C and 37°C. However, oTIS detected a stronger TIS signal (lanes 5 vs 6 and 13 vs 14) while primer oP detected a weaker P signal (lanes 5 vs 6 and 16 vs 17) at 37°C compared to 22°C conditions. In the primer extension reactions oTIS+oP, we estimated a TIS/P ratio of 0.3 at 22°C (lane 5) and 4.4 at 37°C (lane 6) signifying ~15-fold change of the TIS/P rate in plants at 37°C. Remarkably, in the heat-treated seedlings, primer extension with oTIS also revealed the accumulation of products mapping at +10/+11, +20/+21, +29/+30, +38/+39/+40, and +47 relative to the TIS (lane 14, black arrows). Multiple signals upstream of the P site also accumulate in the heat-treated seedlings (lane 10, vertical dashed arrow) and likely come from accumulating pre-rRNAs non-cleaved at the P site, including the 45S.

Together these results show that heat stress does not de-repress 45S rDNA expression from inactive NOR2. In contrast, heat stress provokes accumulation of rRNA transcripts from active NOR2. Transcripts accumulating at 37°C are accurately initiated from +1 whereas accumulation of transcripts cleaved at the P site decreased at 37°C.

### Heat stress inhibits accumulation of pre-rRNA from major and minor processing pathways

**Figure 3.**
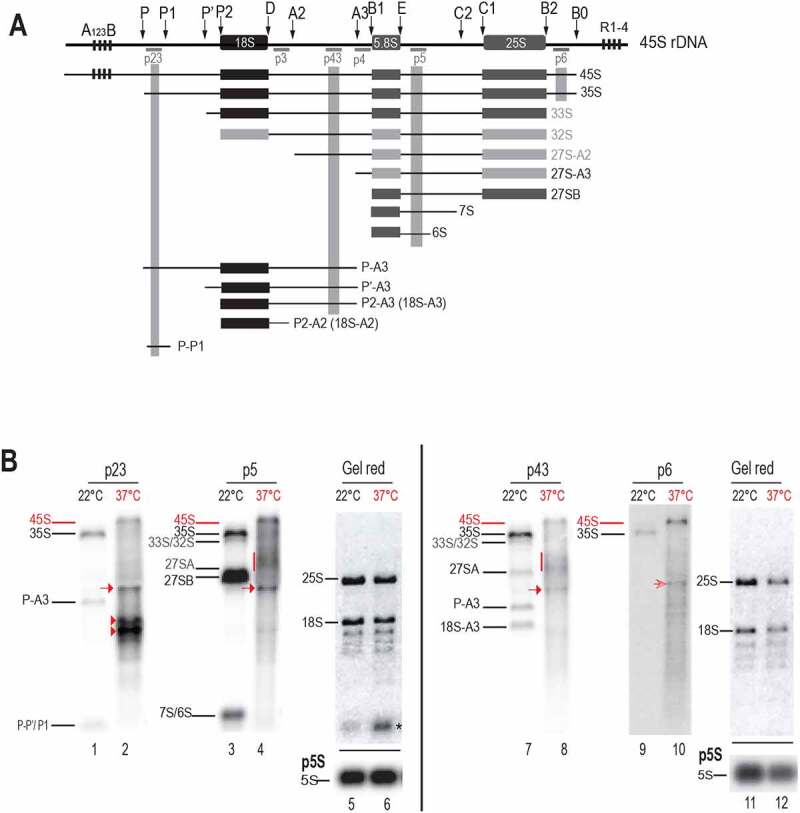
Pre-rRNA processing in response to heat stress. A) Scheme representing 45S rDNA and pre-RNA transcripts detected with probes hybridizing 5’ETS (p23), ITS1 (p43), ITS2 (p5), and 3’ETS (p6) sequences. rRNA precursors and fragments from major ITS1-first (black labelled) and minor 5’ETS-first (grey labelled) pathways are illustrated. B) Northern blot analysis of total RNAs from non-treated (even lanes) and heat treated at 37°C during 24 h (odd lanes) seedlings. A first membrane was hybridized with p23 and p5 (lanes 1–4) and a second one with p43 and p6 (lanes 7–10). Detected pre-rRNAs are labelled accordingly to previous reports using same probes. Pre-rRNAs detected upon heat stress are red labelled. Both membranes were stained with Gel Red to verify quality and amount of RNAs from samples at 22°C (lanes 5 and 11) and 37°C (lanes 6 and 12) and to localize the relative position of rRNAs 18S and 25S. The asterisk in lane 6 indicates a sporadic and unknown RNA species. RNA amounts for each sample were also verified by hybridization with p5S to detect 5S rRNA.

As heat stress treatment provokes altered accumulation of 45S (TIS signal) and 35S (P signal) pre-RNAs, we examined if major ITS1-first and minor 5 ETS-first rRNAs processing pathways [4,7,16] were also affected in seedlings maintained at 37°C for 24 h and in plants returned to optimal growing conditions after heat stress (Fig. 3).

We performed Northern blot experiments with oligonucleotide probes p23, p5, p43 and p6 (Table S1), to assess pre-rRNAs accumulation and cleave-off products in *Arabidopsis thaliana* plants. In wild-type Col-0 plants these primers are known to detect pre-rRNAs from the major ITS1-first pathway, such as 35S, 27S-A3, 27SB, P-A3, P’-A3, 18S-A3, 7S/6S and 5’ETS cleave-off P-P’/P1. They are also able to detect 5’ETS pre-rRNAs from the minor 5’ETS-first pathway: 33S/32S 27S-A2 (Fig. 3A and [13,14,35]). Therefore, under optimal temperature conditions (22°C), these primers detected pre-rRNAs from the major ITS1-first pathway and from the minor 5’ETS-first pathway (Fig. 3B, lanes 1, 3, 7 and 9). Remarkably, in the seedlings exposed to heat stress (37°C), pre-rRNAs from the major ITS1-first pathway 35S, P-A3, 18S-A3, 7S/6S and fragment P-P’/P1 are not detectable; neither pre-rRNAs from the minor 5’ETS- first pathway 33S/32S and 27S-A2 (Fig. 3B, lanes 2, 4, 8 and 10). In contrast, a signal that might correspond to the 45S, the largest expected pre-rRNA in Arabidopsis, is detected with all probes at 37°C (red bar indicated). Diffuse rRNA intermediates (red vertical line), migrating below 33S/32S and above 27SA are detected as well with p5 and p43 (lanes 4 and 8) but not with p23 (lane 2) or p6 (lane 10), indicating that 5’ETS and 3’ETS of these intermediates are removed. Interestingly, all four probes also detected the accumulation of a specific pre-rRNA intermediate migrating below 27SB and above P-A3 (lanes 2, 4, 8, and 10, red arrows). An approximate estimation of the length of these transcripts is approximately 3 kb. In addition in the heat-treated seedlings, p23 specifically detected two strong signals migrating below the P-A3 at ~2 kb (lane 2, red arrowheads). These ~2 kb transcripts are not detected with any other probe, and they are likely 5’ETS-transcript products with 5’ end at +1 and 3’ end at P2/P’ sites.

**Figure 4.**
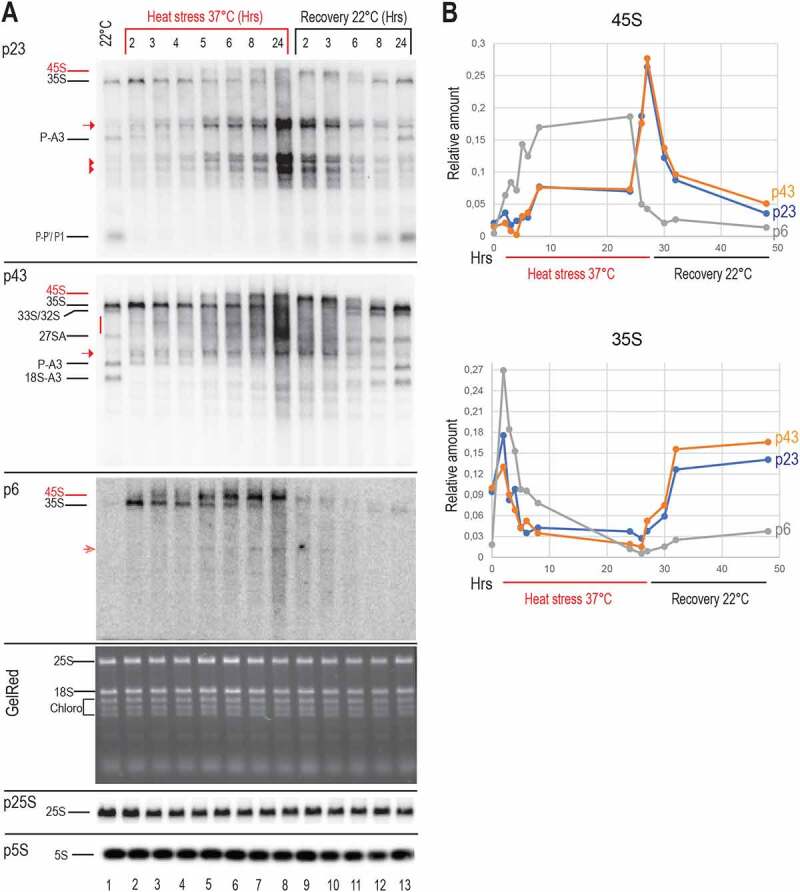
Kinetics of pre-rRNA processing throughout heat stress and recovery conditions. A) Northern blot analyses of total RNAs from non-treated (lane 1), heat treated (lanes 2–8), and recovered (lanes 9–13) seedlings. The same membrane was hybridized with p23, p43, and p6 probes, and similar RNA amounts for each sample were verified by Gel Red staining and hybridization with p5S and p25S to detect respectively 5S and 25S rRNAs. Pre-rRNAs detected upon heat stress are red labelled. B) Graphs show accumulation of pre-rRNAs 45S and 35S during heat (red underlined) and recovery (black underlined) periods. Relative amounts of each pre-rRNA detected with *p23*, p43 and *p6* in each lane were determined, normalized to 5S rRNA signals (Table S5) and represented in blue, Orange and grey respectively.

Next, we determined the kinetics of pre-rRNAs upon heat stress (37°C for 2 h, 3 h, 4 h, 5 h, 6 h, 8 h and 24 h) and in seedlings returned to optimal growth conditions after heat treatment (R22°C for 2 h, 3 h, 6 h, 8 h, and 24 h) (Fig. 4A). Signals corresponding to 35S species progressively decreased in the course of heat stress (p23, p43 and p6; lanes 3–8) while P-A3, 18-A3 and P-P’/P1 transcripts completely disappeared after 2 h-3 h at 37°C (p23 and p43 lanes 2 and 3). In contrast, 45S and other heat stress-specific pre-rRNAs previously identified (Fig. 3) progressively accumulated from 2 h to 24 h of heat stress (p23, p43, and p6, red arrows, arrowheads and red dash). When the heat-treated seedlings were returned to 22°C (Recovery 22°C), the pre-rRNA profiles were progressively restored to those observed in non-treated seedlings (22°C, lane 1). Indeed, 45S signal decreased after 3–6 h of recovery while 35S, P-A3, 18S-A3 (and likely co-migrating P’-A3, 18S-A2) pre-rRNAs, and P-P’/P1 fragments were detected again after 6 h-8 h of recovery (p23, p43, and p6, lanes 9–13). Similar results for P43 were obtained upon heat stress for 6 h, 18 h, 24 h and 30 h at 37°C and in seedlings returned to optimal growth conditions (R22°C for 6 h, 12 h, 24 h, and 50 h) after heat treatment (Figure S4 and S5).

Then, we determined relative amounts of 45S and 35S detected at each point of the kinetic (Fig. 4B). The graphical representation shows progressive reduction of 35S while 45S accumulated at 37°C. During the recovery phase at 22°C pre-rRNA profiles were progressively restored near to those of non-stressed plants. Noticeably, the 45S and 35S transcript signals detected by p6 accumulated relatively faster at 37°C when compared to p23 and p43 signals, while the 35S transcript level recovered much slower after returning to 22°C. This might indicate that p6 detect 3’ETS tailed 45S and 35S pre-rRNA while p23 and p43 might detect 45S and 35S -pre-rRNAs with or without 3’ETS sequences.

Altogether the data revealed that pre-rRNA processing, and specifically the major ITS1-first and the minor 5’ETS pathways, is compromised in Arabidopsis seedlings exposed to 37°C, in a reversible manner. Northern blot results also indicate that heat stress induces early accumulation of specific rRNA transcripts or products, including 45S pre-rRNAs and 5’ETS-products.

### Trimmed 5’ETS and P-cleaved rRNA products accumulate during heat stress

**Figure 5.**
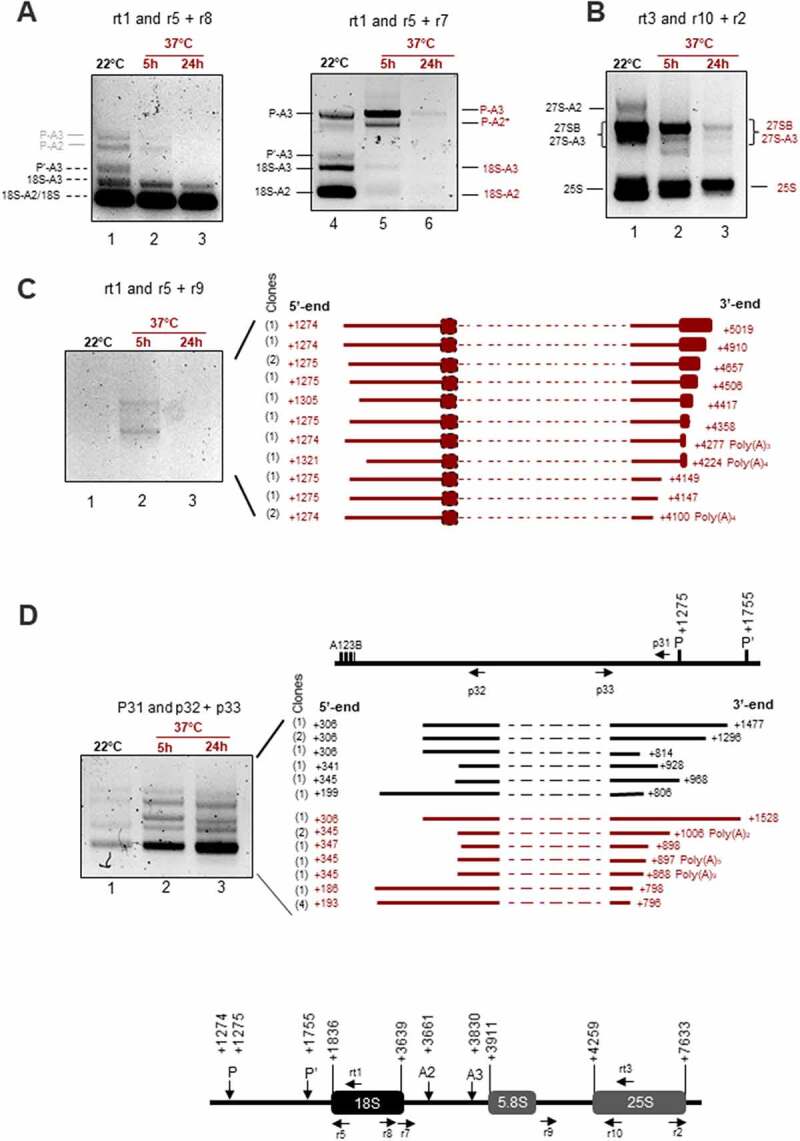
cRT-PCR amplifications to detect rRNA transcripts and products upon heat stress. The upper schemes represent a 45S rRNA sequences to show positions of primers used for cRT-PCR amplifications. cRT-PCR was performed on circularized RNAs from non-treated (22°C) or heat treated (37°C for 5 h or 24 h) seedlings with primers rt1, rtt3, or p31 for RT, and **A)** primers pairs r5+ r8 and r5+ r7, **B)** r10+ r2, **C)** r5+ r9, and **D)** p32+ p33 for PCR amplifications. Circular RT-PCR amplification products detected at 22°C and 37°C and identified by sequencing are respectively represented in black and in red. cRT-PCR products deduced by sizes are labelled in grey. The products sequenced after cRT-PCR reactions with rt1/r5+ r9 and p31/p32+ p33 are represented. Given that the primers used are divergent, part of the cDNA obtained from each circularized rRNA transcript (dotted lines) is absent from the final circular RT-PCR products. For each rRNA transcript, the 5’ and 3’ end positions are indicated. The number of sequenced clones are indicated on the left between brackets.

Northern blot experiments have revealed a rapid and substantial decrease of pre-rRNA precursors of 18S, 5.8S and 25S rRNAs, whereas the 45S, 5’ETS products and an unknown rRNA transcript accumulated after heat stress treatment. To better characterize rRNA transcripts accumulating at 37°C, we performed circular PCR amplifications (cRT-PCR), using specific RT and PCR primers (Table S1) and total RNAs from control non-treated (22°C) and heat treated (37°C for 5 h and 24 h) seedlings. Then, single bands or total PCR products were cloned and sequenced for identification (Fig. 5).

First, cRT-PCR with primer rt1 for RT from 18S, and primer couples r5+ r7 or r5+ r8 for PCR amplifications (Fig. 5A) identified P-A3, P’-A3, 18-A3, 18-A2 and 18S in the control non-treated plants (lanes 1 and 4) and P-A3 and P-A2* after 5 h at 37°C (lanes 2 and 5). None of these precursors, excepting mature 18S, were detected after 24 h at 37°C (lanes 3 and 6). Second, cRT-PCR with rt3 for RT from 25S and PCR primers r2+ r10 identified 27S-A2, 27S-A3, 27SB and 25S (Fig. 5B). Higher accumulation of all these transcripts was detected in the cRT-PCR reactions with RNA from non-treated (lanes 1) compared to heat-treated seedlings for 5 h and 24 h (lanes 2 and 3). Interestingly, the cRT-PCR reactions detected similar amount of 18S rRNA at 22°C and 37°C while amplification of 25S rRNA decreased after 5 h and 24 h of heat stress. These conditions of cRT-PCR also allowed mapping of 5’ and 3’ ends of 18S (1836–3639); 5.8S (3911) and 25S (4259–7633), as well as P (1274 and 1275), P’ (1755), A2 (3661) and A3 (3830) sites in our experimental plant growing conditions (Table S2). Third, in order to detect long pre-RNAs that might accumulate specifically at 37°C and may correspond to those detected by Northern blot (indicated by red arrows, arrow heads or vertical dash), we performed cRT-PCR with RT primer rt1 and PCR primers r5+ r9 (Fig. 5C). We detected two major pre-rRNAs intermediates after 5 h (lane 2) but not after 24 h (lane 3) at 37°C or control (lane 1) seedlings. An aliquot of the PCR reaction (lane 2) was used to clone all rRNA transcripts. We sequenced 13 different rRNA clones. The 5’ end of 11 rRNAs clones is located at +1274 or +1275 (P site), while for 2 rRNA clones, the site was mapped at +1305 and +1321. The 3’ ends were more variable: they were mapped from +4100 in the ITS2 to +5019 in the 25S. Noticeably, two rRNA sequences, ended at +4100 and +4277, have three and four adenosines not detected in the 45S rDNA reference sequence (available in supplementary Information 1). Finally, to identify 5’ETS rRNA transcripts or products, we performed cRT-PCR reactions with RT primer p31 located upstream of P site and PCR primers p32+ p33 (Fig. 5D). We detected cRT-PCR products with similar sizes at 22°C and 37°C. However, these rRNA products accumulated after 5 h and 24 h at 37°C (lanes 2 and 3) in contrast to 22°C (lane 1). An aliquot of these PCR reactions was used for cloning and identification of rRNA transcripts. We obtained 7 and 11 clones, respectively, from 22°C and 37°C samples. At 22°C (black labelled) we detected 5’ETS rRNA species with 5’ ends at positions downstream of A^123^B boxes (from +199 to +306) and with 3’ ends upstream of P and P’ sites (from +806 to +928) sites. At 37°C (red labelled), the 5’ ends were detected also downstream of A^123^B boxes (from +186 to +347) and with 3’ ends upstream of P and P’ sites (from +796 to +1006).

Together, these data indicate that heat stress inhibits accumulation of canonical pre-rRNAs while 3’-trimmed P-cleaved pre-rRNAs and 5’ETS fragments accumulated transiently (5 h) or more stably (5 h-24 h) at 37°C.

### *Accumulation of 5’ETS rRNA products is inhibited in* nuc1-2 *plants at 37°C*

**Figure 6.**
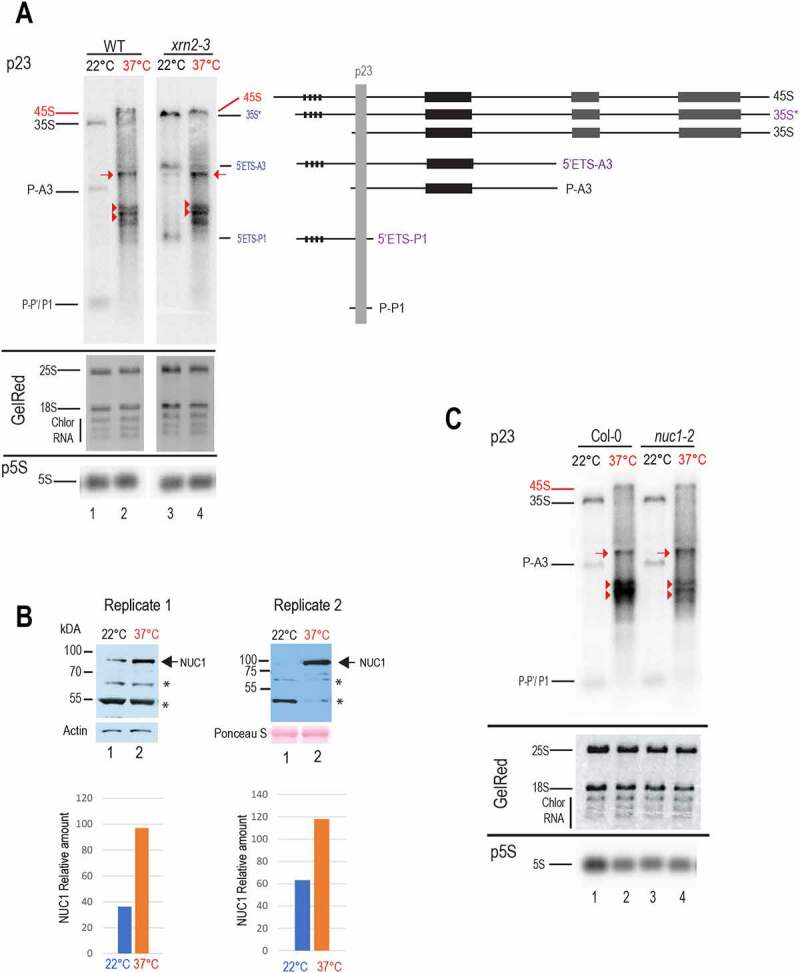
Pre-RNA processing in *xrn2-3* and *nuc1-2* in response to heat-stress. A) Northern blots analysis of total RNAs from Col-0 Arabidopsis WT and *xrn2-3* seedlings non-treated (22°C, lanes 1 and 3) and heat-treated (37°C for 24 h, lanes 2 and 4) using probe p23. pre-rRNA transcripts 35S, P-A3 and fragments P-P’/P1 detected in WT are indicated in black and 35S*, 5’ETS-A3 and 5’ETS-P1 specifically detected in *xrn2-3* [11] are in purple. The rRNA transcripts detected upon heat stress are red labelled: 45S, arrows, and arrowheads. Similar amounts of RNAs in each sample were verified by Gel Red staining. **B)** Western blot analysis of total protein extracts from two biological replicates of non-treated (lanes 1) and heat treated (37°C) for 24 h (lanes 2) seedlings. α-NUC1 antibody detects NUC1 protein (arrow) and two unspecific protein bands (asterisks, and see Figure S6B). Actin and Ponceau S are used as loading controls. The bar graphs show the relative amount of NUC1 in each Western blot. α-NUC1 and α-actin signals were quantified with ImageJ, and the amount of NUC1 normalized to actin values (Table S3). **C)** Northern blots analysis of total RNAs from Arabidopsis WT and *nuc1-2* seedlings in non-treated (22°C, lane 1 and 3) and heat-treated (37°C for 24 h, lanes 2 and 4) using probe p23. Pre-rRNAs detected specifically in the heat stressed plant samples are indicated in red: 45S, arrows, and arrowheads. Similar amounts of RNAs in each sample were verified by Gel Red staining and hybridization with p5S to detect 5S rRNA.

In *Arabidopsis thaliana*, 5’-3’ exonucleolytic trimming of the 5’ETS by AtXRN2 is required to expose the site P for subsequent endonucleolytic cleavage [11]. In crucifer plants, a nucleolin-U3 snoRNP protein complex was shown to reproduce accurate cleavage at the P site *in vitro* [5]. Nucleolin is an abundant nucleolar rRNA binding protein required for primary cleavage in yeast, mammals, and plants [10,36–38]. We examined the role of AtXRN2 and NUC1 proteins in pre-rRNA processing in plants upon heat stress (Fig. 6).

Firstly, we investigated whether the accumulation of specific rRNA transcripts and products detected at 37°C relies on XRN2 activity. Arabidopsis Col-0 and *xrn2-3* plants were thus maintained at 22°C or heat stressed at 37°C for 24 h, and the accumulation of pre-rRNAs assessed by Northern blot (Fig. 6A). Under optimal growing conditions, the *xrn2-3* mutant plants display atypical 35S*, 5’ETS-A3 and 5’ETS-P1 rRNA transcripts [11]. As expected these rRNAs were detected in the *xrn2-3* (lane 3) but not in the control Col-0 (lane 1) plants at 22°C. In contrast, the pre-rRNAs detected at 37°C in *xrn2-3* (lane 4) matched those observed in Col-0 (lane 2). It is also noticeable that the 5’ETS heat stress species (~2 kb rRNAs) in Col-0 and in *xrn2-3* (lanes 2 and 4) are not the 5’ETS-A3 or 5’ETS-P1 fragments detected in *xrn2-3* plants (lane 3) since they migrate below or just above of these 5’ETS- species.

Then we studied NUC1 protein expression and how the absence of NUC1 might affect accumulation of 45S, 35S and ~2 kb 5’ETS products under heat stress conditions. We first performed Western blot analysis of protein extracts prepared from two independent samples of non-treated (22°C) and heat-treated (37°C, 24 h) seedlings (Fig. 6B). Hybridization with antibodies α-NUC1 detected significant accumulation (~1.8- and ~2.8- fold, Table S3) of NUC1 protein in protein extracts from heat-treated (lanes 2) compared to non-treated (lanes 1) seedlings for both replicates. Noticeable, the bands present at a similar level in both samples (asterisks) are also detected in *nuc1-2* mutants and are therefore non-specific signals (Figure S6A and [31]). Since NUC1 interacts with U3 snoRNP [5], we also verified the expression of fibrillarin and U3 snoRNA in response to heat stress. Primer extension with probe pU3 (Table S1) showed no significant change in the amount of U3 snoRNA after heat treatment compared to control plants. In contrast while Fibrillarin protein was downregulated, FIB1 and FIB2 transcripts accumulated at 37°C (Figure S6B and C). Then, to determine how the absence of NUC1 might affect accumulation of 5’ETS transcripts we performed a Northern blot with probe p23 to determine accumulation of pre-rRNAs at 22°C and 37°C in *nuc1-2* mutant plants (Fig. 6C). We observe a similar accumulation of 35S, P-A3, and P-P1 in Col-0 and *nuc1-2* seedlings at 22°C (lanes 1 and 3). Similar amounts of 45S and ~3 kb transcripts (red arrows) were also detected at 37°C (lanes 2 and 4). Interestingly at 37°C, we detected much less ~2 kb 5’ETS transcripts in the *nuc1-2* than in Col-0 plants (lanes 2 and 4, red arrowheads).

Taken together, our results indicate that the absence of XRN2 and/or accumulation of specific pre-rRNA transcripts and 5’ETS fragments in *xrn2-3* mutants does not affect overall rRNA transcript accumulation of Arabidopsis plants exposed to heat stress conditions. In contrast, upregulation of NUC1 protein expression at 37°C seems to be linked to an increased amount of aberrant 5’ETS species in response to heat stress.

### Heat stress provokes ribosome profile changes

**Figure 7.**
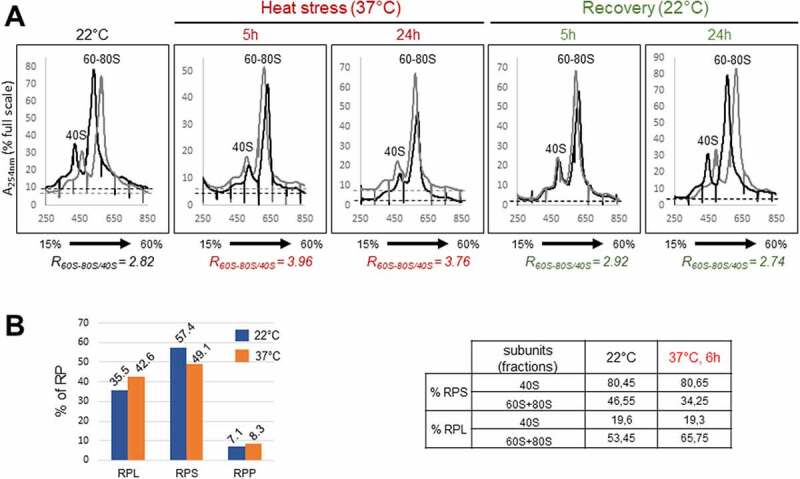
Ribosome profiles and LC-MS/MS analysis on ribosome subunits in response to heat stress. A) Extracts from two experimental replicates (black and grey) of non-treated (22°C), heat treated (37°C for 5 h and 24 h), and recovered (37°C for 24 h then 22°C for 5 h and 24 h) seedlings were fractionated through 15–60% sucrose gradients. The percentage of the full scale of absorbance was monitored at 254 nm. Peaks corresponding to 40S and 60S-80S ribosomal subunits/monosomes are indicated and controlled by the presence of mature 18S and/or 25S rRNAs (Figure S7). The ratios of the 60–80S over the 40S were calculated for each replicate at each temperature, and the average Ratio (R_60-80/40S_) for each condition is indicated below. **B)** LC-MS/MS of ribosome subunits 40S and 60S-80S from sucrose gradient from non-treated (22°C) and 6 h, 37°C heat treated plants (Figure S7). Left Panel, relative amount (in %) of RPS, RPL and RPP in the 40S+60S+80S peaks at 22°C (fractions 8–11) and 37°C (fractions 9–12). Right panel, relative amounts of RPS, RPL and RPP in the 40S at 22°C (fractions 8 + 9) and (fractions 9–10) at 37°C or in the 60S-80S at 22°C (fractions 10 + 11) and at 37°C (fractions 11–12).

As heat stress induces nucleolus disorganization and inhibits accumulation of pre-rRNA precursors of 18S, 5.8S and 25S, we wondered if these modifications could affect the ribosome assembly. To do so, we investigated ribosome profiles in heat-treated and recovered seedlings. Whole-cell extracts from Arabidopsis seedlings non-treated (22°C), heat treated (37°C for 5 h and 24 h) and recovered (R22°C for 5 h and 24 h) were fractionated through a sucrose cushion to remove plastid ribosomes [39], then through a 15–60% sucrose gradient to separate cytoplasmic 40S and 60S ribosomal subunits and 80S monosomes from polysomes. Results from two independent experiments (for each temperature condition) are presented on single graphs (Fig. 7 and Table S4). In all extracts from non-treated (22°C), heat treated (37°C 5 h and 24 h), and recovered (R22°C, 5 h and 24 h) seedlings, the 40S was clearly resolved, whereas the 60S particles sedimented with the 80S. To estimate accumulation changes, we then calculate the ratio of 60S-80S over 40S (R_60S-80S/40S_). While the average ratio from two replicate experiments is 2.82 (see values in Table S4) at 22°C, this ratio drastically increases up to 3.96 and 3.76 after 5 h and 24 h at 37°C, respectively. Remarkably, the ratios for the 5 h and 24 h recovered seedlings are 2.92 and 2.74, near to values observed in non-treated seedlings.

To better understand ribosome profile changes observed at 37°C we determined relative amounts of ribosomal proteins in peaks 40S and 60S-80S. For this analysis, we carried out additional gradient fractionations (Figure S7). Note that in these experiments, the ratio R_60S-80S/40S_ also increases from 2.5 at 22°C to 4.75 at 37°C, which is consistent with increased values obtained in the experiment shown in Fig. 7A. Then, we performed LC-MS/MS on 40S and/or 60–80S peak fractions (Fig. 7B, S4 and Supplementary Data 1). First, this analysis revealed that amongst all the spectra associated to ribosomal proteins, the proportions (%) of RPL, RPS and RPP proteins in the 22°C plant fractions (fractions 8–11) were, respectively, 35.5%, 57.4% and 7.1% while in the heat treated plant fractions (fractions 9–12), the proportion of RPL, RPS and RPP proteins was, respectively, 42.6%, 49.1%, and 8.3% (7B, graph left panel). This represents a slight decrease of RPS, and an increase of RPL and RPP proteins in heat stressed (37°C) compared to control (22°C) plants. Then, we determined the distribution (% of spectra) of RPS and RPL+RPP proteins in the 40S (fractions 8 + 9 at 22°C and 9 + 10 at 37°C) and 60S-80S (fractions 10 + 11 at 22°C and 11 + 12 at 37°C) peak fractions (Fig. 7B, Table right panel). In the 40S fractions at 22°C, we found ~80% of RPS and ~20% of RPL+RPP. Noticeably, similar values were estimated at 37°C. In contrast, in the 60S+80S fractions, different values were obtained: at 22°C we estimated ~47% of RPS and 53% of RPL+RPP, while ~34% of RPS and 66% of RPL+RPP were estimated at 37°C.

In conclusion, we showed that heat stress impairs the accumulation of 40S and/or favours the accumulation of 60S-80S ribosome subunits/monosomes, in a reversible manner. Furthermore, the LC-MS/MS analysis showed that under heat stress the protein level of ribosomal proteins in the 40S and 60S-80S fractions is slightly downregulated (RPS) or up-regulated (RPL+RPP). This analysis also revealed that RP ratio changes in the 60S+80S fractions but not in the 40S fractions upon heat stress, and thus suggest increased 60S and/or 80S particles.

## Discussion

In this work, we showed first that a long and permissible heat stress treatment provoked rapid and reversible changes of nucleolus morphology in *Arabidopsis thaliana*. In contrast, the reassembly of the nucleolus is relatively slower, compared to the initial heat stress response, after stressed plant seedlings are returned to optimal growing conditions (Fig. 1, S1 and S2). Thus, under heat stress conditions, Arabidopsis plants might not only rapidly down-regulate transcription/processing of pre-rRNAs and assembly of ribosomes, which occur essentially in the nucleolus [28,40], but also other activities linked and/or associated to nucleolus structure [21,41–43]. Correspondingly, plants also sense favourable environmental conditions and progressively allow the reassembly of nucleoli and subsequently ribosome synthesis and other non-ribosome related activities.

Heat stress is known to inhibit rDNA transcription in animal cells [23,44], whereas in Drosophila, heat shocks increase RNA pol I transcription of retrotransposons located in rDNA clusters [45]. In Arabidopsis plants, we showed that heat stress does not release the silencing of inactive rDNA copies from NOR2 [46,47]. In contrast, 45S rRNA gene transcription from active NOR4 might still occur under heat stress leading to the accumulation of 3’ETS rRNA sequences (Fig. 2A) from long pre-rRNA, and not merely cleave-off product[48]. Although it is well established that nucleolus assembly and structure are linked to transcription and processing of pre-rRNA, under heat stress conditions, some rRNA synthesis and maturation seem to be still occurring in disrupted nucleoli in plants (Fig. 2). This is the case in yeast when an intact nucleolus might not be absolutely required for pre-rRNA processing under certain conditions [49].

Notably, heat stress induced the accumulation of primary 45S rRNAs with concomitant reduction of 35S pre-rRNAs (Figs. 3 and 4), indicating that primary cleavage site at P is impaired at 37°C. Consistently, pre-rRNAs initiated from TIS increased while signals from pre-rRNAs cleaved at P site decreased (Fig. 2). Accumulation of 45S and other pre-rRNAs non-cleaved at P site is also supported by increased primer extension signals upstream of P site. In fact, the 1 kb insertion sequence located upstream of the P site contains several repeated sequences [7] that might form stable secondary RNA structures and obstruct RT elongation reactions of these non-cleaved P-site transcripts. Interestingly, rRNA transcripts initiated just downstream of TIS and under heat stress conditions (Fig. 2) might correspond to atypical rRNA transcripts rather than 45S pre-rRNA trimmed by XRN2 [11]. None of these transcripts were detected by cRT-PCR (Fig. 5) consistently with the fact that RNA polymerases use triphosphate nucleotides when initiating transcription, inhibiting the circularization of primary transcripts. Thus, we cannot exclude that ectopic RNA Pol I transcription contributes to the accumulation of atypical 45S pre-rRNA under heat stress conditions, as RNA Pol I might transcribe IGS sequences from cryptic promoters in Arabidopsis [30]. It is neither excluded that RNA pol II might also transcribe rDNA under heat stress conditions as reported in *Candida albicans* during nutritional depletion or TOR inhibition [50]. In normal conditions, RNA pol II is excluded from the nucleolus, but nucleolar disruption at 37°C (Fig. 1 and S2) might enable access of Pol II to transcribe rRNA genes and subsequently initiate transcription from RNA pol I promoters in a non-nucleolar environment [51].

Remarkably, accumulation of rRNA species from major (ITS1-first) and minor (5’ETS-first) processing pathways was rapidly inhibited upon heat stress and can be correlated with rapid changes in the nucleolus morphology. The re-establishment of rRNA precursors to normal profiles is concomitant with the reassembly of the nucleolus when stressed Arabidopsis plant seedlings are returned to optimal conditions (Figs. 1 and 4). In contrast, upon heat stress Arabidopsis seedlings accumulated atypical ~3 kb rRNA transcripts as well as ~2 kb 5’ETS-species (Figs. 3, 4 and 6). Our results support the possibility that accumulation of canonical pre-rRNA is inhibited under heat stress without production of novel pre-rRNA counterpart species. This is also observed in mutant *nuc2-2* plants, displaying expression of specific rRNA gene variants, *rh10-1*, and *rrp7* showing accumulation of pre-RNA from the ITS1 pathway [14,25,52] and *rtl2* defective in RNase III cleavage in the 3’ETS [11,33]. In all mutant plants, we detected at 37°C pre-rRNAs accumulation changes similar to those observed in Col-0 plants (Figures S3 and S9).

In agreement with our results, it was recently reported that 1 h exposure at high temperatures (from 30 to 42°C) provoke significant changes in the accumulation of pre-rRNAs 35SA^123^B/35S*, 35S, 32S, 27S, P-A3, 18-A3 and P-P1 in rosette leafs from Arabidopsis plants [53]. Indeed, in *Arabidopsis* seedlings a rapid reduction of 27SA, P-A3, 18-A3 and P-P1 was also detected after 2 h at 37°C while 35S pre-rRNA levels increased to decrease gradually after 3 h at 37°C (Fig. 4). Besides, the P-C2 rRNA transcripts identified by Shanmugam et al. after 60 and 90 minutes at 38°C, might correspond to the ~3 kb rRNA transcripts. Despite these similarities occurring after 1–2 h of heat stress; we have found specific pre-rRNA changes that might be linked to the duration of heat exposure. This is the case for the 45S and ~2 kb ETS species detected after 3–5 h at 37°C (Figs. 3, 4, 6 and S3-5, S9), and not reported by Shanmugam and colleagues after 60–90 minutes of heat (up to 42°C) treatment [53].

Transient accumulation of transcripts cleaved at the P site and 3’-5’ trimmed from 25S sequences (Fig. 5B) indicate as well that upon heat stress conditions 35S pre-rRNAs (and eventually other P-cleaved pre-rRNAs) are degraded predominantly by the 3’-5’ exonuclease activities rather than being cleaved at alternative sites in the internal or external spacers. This is in contrast to the non-productive pathway described in yeast, where pre-rRNAs produced after an A3 cleavage instead of an A2 cleavage are degraded by the exosome [17]. In a preliminary modelling study addressed to determine processing rates of major 45S, 35S and P-A3 pre-rRNAs (Figure S8, Supplementary Information S2 and Table S5) we showed that heat stress is associated with a 30–90% reduction in the 45S processing rate into 35S (k45S), which leads to the 35S level decrease. Meanwhile, heat stress reduces (35%–36%) the processing of such 35S pre-rRNAs into P-A3, and concomitantly correlates with an 2–3 fold increase in the rate of P-A3 processing/degradation. Notably, we have found a transformation rate (k^s−1^) of 35 into P-13 (α*k_35S_) of (3.6–25)x10^−5^, which is similar in order of magnitude to the k_4_ (2.4 ± 0.4) x10^−5^estimated by pulse-labelling experiments with heat stress at 38°C [53]. Accordingly, these estimations suggest that significant changes in the processing rates of specific rRNAs are required to impair ITS1-first (and likely 5’ETS-first) once plants are exposed to heat stress conditions.

Altogether, we can conclude that coordinated 45S transcription from +1 and initial processing at the P site to generate 35S play major roles in directing productive processing of pre-rRNAs to mature 18S, 5.8S and 25S rRNAs, through the main ITS1-first (and 5’ETS- first to some extent) pathway. In contrast, transcribed pre-rRNAs (from +1 or ectopic TIS) under heat stress conditions are poorly cleaved at P site but still might generate 35S or longer pre-rRNA transcripts, considering that processing of pre-rRNAs in Arabidopsis is post-transcriptional [1,7]. However, these heat stressed 35S pre-rRNAs are degraded by the exosome rather than being cleaved at sites A3 in the ITS1 or P’ in the 5’ETS.

While P- cleaved pre-RNAs (like 35S) seem to be degraded under heat stress, a strong accumulation of 5’ETS transcripts was detected at 37°C (Figs. 3, 4 and 6). These transcripts are not heat-specific since similar 5’ETS products are detected at 22°C (Fig. 5D). These transcripts are likely 5’ETS cleave-off products, cleaved at P sites [11] or 5’ETS-P’ degradation intermediates [54]. Accumulation of 5’ETS-produts at 37°C suggest that their degradation by the exosome is inhibited under heat stress conditions. In contrast, the P-P’ fragments are undetectable after just 2 h of heat treatment, which is in agreement with P cleaved transcripts (35S) being preferentially trimmed by the exosome at 37°C and not cleaved at P’ site or other sites in the ITS1 or ITS2 (Fig. 5B). The exonucleases involved in pre-rRNA processing are relatively well characterized in Arabidopsis. Notably, it is known that 5’ETS fragments cleaved at the P site are not accessible to 5’-3’ exoribonucleases but are predominantly degraded by polyadenylation assisted 3’-5’ RRP6L2 and DIS3/RRP44A exoribonucleolytic decay [11,54–58]. Thus we cannot exclude that polyadenylation of 5’ETS-P(P’) could be also inhibited at 37°C. Arabidopsis TRL, RRP41, and RPP6L2 transcript levels increased at 37°C whereas DIS3/RRP44A transcript levels significantly decreased in response to heat stress (Figure S10 and Table S6). In contrast, transcript levels of 5’-3’ exoribonuclease XRN2 or XRN3 are not significantly altered upon heat stress. We do not know yet if these transcript level variations are associated with protein changes but it would be interesting to further study pre-rRNA in mutant plants for exoribonucleases under heat stress conditions.

The precise nature of the ~2 kb 5’ETS rRNA species accumulating at 37°C remain unknown. They are not the atypical 5’ETS-A3 or 5’ETS-P1 species reported in *xrn2-3* plants mutants (Fig. 6A). However, accumulation of these ~2 kb 5’ETS rRNA species can be linked to a higher amount of NUC1 protein at 37°C (Fig. 6B). Similarly, the low-temperature conditions increased nucleolin NRS1 protein level and led to pre-rRNA processing defects in yeast [38]. Under optimal growth conditions, the absence of NUC1 provokes nucleolus disruption and affects accumulation of rRNA transcripts initiated at the TIS and cleaved at the P site in *nuc1*-2 mutants [10]. Interestingly, *nuc1-2* mutants exposed to heat stress accumulated 45S and ~3 kb rRNA transcripts at similar levels than Col-0 plants, whereas the ~2 kb related 5’ETS-species decreased compared to Col-0 plants (Fig. 6B). NUC1 is an RNA binding protein and, accordingly, increased NUC1 protein might somehow protect 5’ETS rRNA species produced at 37°C from maturation or degradation. Besides, NUC1 protein is required for appropriate RNA processing since absence of NUC1 reduces 2-*O*-methylation of rRNA in *nuc1-2* plants [59]. It is also worth to mention that Arabidopsis encodes a second nucleolin protein gene (NUC2), upregulated in *nuc1*-2 mutants [10]. However, NUC2 is not involved in pre-rRNA processing of P site, at least at 22°C [60].

In addition to altered processing of pre-rRNA, the expression of several ribosome biogenesis and assembly factors are affected as well upon heat stress. Indeed, 149 RP (Ribosomal Proteins) and 77 RBF (Ribosome Biogenesis Factors) genes are differentially expressed after heat stress (Figure S10 and supplementary tables S7-9). Interestingly, *RP* and *RBF* transcripts accumulate under heat stress, suggesting the assembly of specific ribosomes upon heat stress or alternatively the establishment of a mechanism to restore ribosome titre as soon as plants are returned to favourable conditions.

Ribosomes from Arabidopsis are relatively stable, with only half of the ribosome population replaced every 3–4 days in cell cultures [61]. It is nonetheless reasonable to think about a negative impact on the stability of ribosomes in plants under prolonged heat stress conditions, as heat stress provoked rapid imbalanced 40S versus 60S/80S ribosome particle ratios that could be restored as observed in recovered plants. This imbalance is likely due to changes in the ratio of RP specifically in the 60S+80S fraction and not in the 40S fractions (Fig. 7 and S7). Nevertheless, we do not exclude that this imbalance can also be provoked simultaneously by the ribosome degradation pathway called ribophagy [62]. Consequently, a restored balance of ribosome particles during recovery is likely a subsequent assembly of new ribosomes, as previously reported in Arabidopsis young seedlings after heat shock [63]. Finally, ribosome heterogeneity also exists in plants (reviewed in [64] and it cannot be excluded that specialized ribosomes can be assembled after a few hours of heat treatment (heat shock) or prolonged exposure to milder temperature. Determining the molecular bases of alternative pre-rRNA processing (including RNA modifications), degradation and assembly of specialized ribosomes during plant development and responding to environmental conditions are the related challenges to be addressed.

## Materials and methods

### Plant materials and growth conditions

All lines were derived from *Arabidopsis thaliana* Columbia (Col-0) ecotype. Mutant lines used in this work were previously described: *xrn2-3* [11], *rrp7-1* [52] and *nuc1-2* [31]. Seeds were first sown on 1X Murashige and Skoog (MS) medium (Duchefa Biochemie M0231), including Gamborg B5 vitamins, and supplemented with 1% Sucrose, 0.05% (w/v) MES, and 1% (w/v) plant agar (pH 5.7). After two days at 4°C, plants were grown for 14 days under a 16 h/8 h photoperiod (light/dark, 22°C/20°C) in Percival growth chambers set with light intensity 180 μE.m-2. s-1 and hygrometry 55%/60% for light/dark, respectively.

For heat stress, 14 days-old seedlings were transferred to Percival chambers set at 37°C for 2 h to 8 h (during the light cycle) or 24 h (16 h/8 h photoperiod). For recovery experiments, seedlings treated for 24 h at 37°C were returned to Percival chambers set at 22°C for 2 h to 24 h (light cycle). Non-treated (22°C), heat treated (37°C) and recovered (R22°C) seedlings were collected, ground to a fine powder in liquid nitrogen with a Retsch MM400 Cryogrinder (frequency 25/s during 30s) and stored at −80°C.

### Transmission Electron Microscopy (TEM)

Roots from non-treated (22°C), heat treated (37°C for 5 h, 8 h, and 24 h) and recovered (R22°C for 24 h) seedlings were fixed with 3% (v/v) glutaraldehyde in 0.025 M Cacodylate buffer pH 7.3 at room temperature. After washing, the samples were post-fixed by 1% OsO4 in the same buffer. The samples were then dehydrated in a methanol series (30%, 50%, 70, and 100%) at room temperature. The samples were acetylated and methylated with a freshly prepared 5:1 (v/v) methanol/acetic anhydride mixture at 25°C. Samples were then washed in pure methanol and embedded in Epon 812 resin (EMS). Ultrathin sections were performed on an ultramicrotome (Leica Ultracut) and counterstained by uranyl acetate and lead citrate before being observed on a 7500 Hitachi TEM [10,65].

### Northern blot and primer extension

Total RNA extractions and 5’ end-labelling of oligo probes (p23, p43, p5 and p6, oTIS, oP, pU3, and pU6; see Table S1) were performed as described previously [31]. Northern blots and primer extension gels were performed using 3 μg and 6 μg of total RNAs, respectively. Northern blot membranes were pre-incubated in PerfectHyb Plus Hybridization Buffer (Sigma) for at least 3 hours at 42°C. Labelled probes p23, p43, p5, and p6 were then added (1 µl at 10 µM) and incubated overnight at 42°C. Membranes were washed at 50°C during 15 min with 2X SSC 0.1% SDS, then with 0,5X SSC, 0.1% SDS, and finally with 0,1X SSC 0.1% SDS. Primer extension and dideoxy sequencing reactions were performed according to [10]. Northern blots and primer extension reactions were analysed on a Personal Molecular Imager (PMI, BioRad) and quantified using Quantity One software.

### Circular RT-PCR and sequencing

Total RNAs (5 μg) from non-treated (22°C) and heat treated (37°C for 5 h and 24 h) seedlings were circularized with T4 RNA ligase 1 (NEB M0204S). Then, the circular RNAs (1 μg) were reverse transcribed with primers p31, rt1, and rt3 to, respectively, hybridize 5’ETS, 18S, and 25S rRNA sequences. cDNAs were PCR amplified with primers r2, r5, r7, r8, r9, r10, p32 and p33 (Table S1). PCR products were cloned into a pGemT easy vector (Promega) and sequenced with primer T7 by Eurofins Genomics (France). Sequences were analysed using reference sequence provided in Supplementary Information 1 and software Geneious 11.0.5.

### Western blot

Total proteins (100 mg) from 15 days-old seedlings non-treated (22°C) and heat treated (37°C, 24 h) were extracted in 500 µL of extraction protein buffer (50 mM Tris-Cl pH 8, 150 mM NaCl, 10 mM EDTA, 50 mM Na fluoride, 1% NP40, 0.45% Na deoxycholate, 1% SDS) supplemented with 20 µM Mg132 (SIGMA) and 1/100 of protease inhibitor Cocktail for Plant cell and Tissue extract (SIGMA). Proteins were then fractionated on SDS-PAGE and analysed by Western blot as previously described in [60]. The membranes were hybridized with a 1:10,000 dilution of α-NUC1 [31], with a 1: 2500 dilution of α-FIB [5] or with a 1:7,000 dilution of α-Actin (Life Technologies). Western blots bands were analysed with Image J (Table S3).

### Sucrose cushions and gradients

All buffers were described in [39] and all steps performed at 4°C. Briefly, 1 to 2 g of frozen powder from non-treated (22°C), heat treated (37°C for 5 h and 24 h) and recovered (R22°C for 5 h and 24 h) seedlings were suspended in 4 mL of PEB, incubated on ice for 30 min and centrifuged 15 min at 16,000 g to remove debris. Samples were then filtered on 0.2 µM filters (Sarsted), loaded on 8 mL sucrose cushions, and centrifuged in a Beckman SW41 rotor for 18 h at 35,000 rpm. Pellets were re-suspended in 1 mL of RB and kept on ice for 30 min. A short centrifugation (2 min at 5,000 g) was performed to remove the last debris. Finally, 1 mL of supernatant was layered onto 9 mL linear 15–60% sucrose gradients and centrifuged at 38,000 rpm for 6h30. Gradients were fractionated using the Type 11 Optical Unit (Teledyne ISCO) system and a UA-6 UV/VIS Detector (Teledyne ISCO) at 254 nm. Values and ratios for 40S and 60S/80S peaks are available in Table S4.

### Liquid chromatography-tandem Mass Spectrometry (LC-MS/MS) analyses

500 µl sucrose fractions obtained from plant samples at 22°C (fractions 8–11) and 37°C (fractions 9–12) were cleaned using a Filter-Aided Sample Preparation (FASP) to remove sucrose and buffer. Five fmol of Bovine Serum Albumin (BioRad, Des Plaines, USA) standard protein were added to each fraction as a positive control of the sample preparation. Proteins were then reduced, alkylated and digested with rapid trypsin/LysC in a 1:10 enzyme:protein ratio (Promega, Madison, USA). Peptides were acidified with formic acid, desalted on a Bravo AssayMap (Agilent Technologies, Santa Clara, USA) and injected on an LC-MS/MS coupling. LC-MS/MS analyses of peptide extracts were performed on a NanoAcquity LC-system (Waters, Milford, MA, USA) coupled to a Q-Orbitrap (Q-Exactive Plus from Thermo Fisher Scientific, Waltham, MA, USA) mass spectrometer equipped with a nanoelectrospray ion source. Database searches were performed using Mascot (version 2.6.2, MatrixScience, London, UK) against an *Arabidopsis thaliana* protein sequences database downloaded from The Arabidopsis Information Resource TAIR site (TAIR10 version gene model), in which common contaminants and decoy sequences were added (2 x27534protein entries in total). Spectra were searched with a mass tolerance of 15 ppm in MS mode and 0.07 Da in MS/MS mode. One trypsin missed cleavage was tolerated. Carbamidomethylation of cysteine residues was set as a fixed modification. Oxidation of methionine residues and acetylation of proteins’ n-termini were set as variable modifications. Identification results were imported into Proline software (http://proline.profiproteomics.fr/) for validation. Peptide Spectrum Matches (PSM) with pretty rank equal to one were retained. False Discovery Rate was then optimized to be below 1% at PSM level using Mascot E-value and below 1% at Protein Level using Mascot score and more than one specific peptide was required. Protein abundances of RPS, RPL and RPP ribosomal proteins were estimated using weighted spectral counts. Detailed sample preparation and LC-MS/MS protocols and data analysis workflows are provided in Supplementary Information 3 and data 1.

## Supplementary Material

Supplemental Material

## Data Availability

Authors responsible for the distribution of material integral for the findings presented in this article is Julio Sáez-Vásquez (saez@univ-perp.fr). All RNA-seq raw data used to generate Figure S10 were submitted to the Sequence Read Archive (SRA), BioProject: PRJNA732814. https://dataview.ncbi.nlm.nih.gov/object/PRJNA834587.

## Note Added in Proof

Since the completion of this work, a relevant paper has appeared: Dynamics and thermal sensitivity of rRNA maturation paths in plants. Shanmugam et al., Journal of Experimental Botany, Vol. 72, No. 21 pp. 7626–7644, 2021. This study supports our findings on rapid and reversible processing changes of pre-rRNA is response to high temperature in Arabidopsis. They also reported similar pre-rRNA processing changes in tomato and rice plants.

## Accession numbers

NUC1 (At1g48920), FIB1 (At5g52470), FIB2 (At4g25630), XRN2 (At5g42540), RRP7 (At5g38720), LAS1-like (At5g12220), MRP1/POP1 (At2g47300), MRP1/POP4 (At2g43190), UTP24 (At2g46230), NOB1 (At5g41190), RCL1 (At5g22100), RTL2 (At3g20420), XRN3 (At1g75660), NOL12 (At1g11240), DIS3/RRP44A (At2g17510), RRP6L2 (At5g35910), RRP41 (At3g61620) and TRL (At5g53770) and MTR4 (At1g59760).

## References

[1] Henras AK, Plisson-Chastang C, O’Donohue MF, et al. An overview of pre-ribosomal RNA processing in eukaryotes. Wiley Interdiscip Rev RNA. 2015;6(2):225–242. 25346433 10.1002/wrna.1269 PMC4361047

[2] Sharma S, Lafontaine DL. ‘view from a bridge’: a new perspective on eukaryotic rRNA base modification. Trends Biochem Sci. 2015;40(10):560–575. 26410597 10.1016/j.tibs.2015.07.008

[3] Sloan KE, Warda AS, Sharma S, et al. Tuning the ribosome: the influence of rRNA modification on eukaryotic ribosome biogenesis and function. RNA Biol. 2017;14(9):1138–1152. 27911188 10.1080/15476286.2016.1259781 PMC5699541

[4] Tomecki R, Sikorski PJ, Zakrzewska-Placzek M. Comparison of preribosomal RNA processing pathways in yeast, plant and human cells - focus on coordinated action of endo- and exoribonucleases. FEBS Lett. 2017;591(13):1801–1850. 28524231 10.1002/1873-3468.12682

[5] Saez-Vasquez J, Caparros-Ruiz D, Barneche F, et al. A plant snoRNP complex containing snoRNAs, fibrillarin, and nucleolin-like proteins is competent for both rRNA gene binding and pre-rRNA processing in vitro. Mol Cell Biol. 2004;24(16):7284–7297. 15282326 10.1128/MCB.24.16.7284-7297.2004 PMC479724

[6] Eichler DC, Craig N. Processing of eukaryotic ribosomal RNA. Prog Nucleic Acid Res Mol Biol. 1994;49:197–239. 7863007 10.1016/s0079-6603(08)60051-3

[7] Sáez-Vásquez J, Delseny M. Ribosome biogenesis in plants: from functional 45S ribosomal DNA organization to ribosome assembly factors. Plant Cell. 2019;31(9):1945–1967. 31239391 10.1105/tpc.18.00874 PMC6751116

[8] Phipps KR, Charette J, Baserga SJ. The small subunit processome in ribosome biogenesis-progress and prospects. Wiley Interdiscip Rev RNA. 2011;2(1):1–21. 21318072 10.1002/wrna.57 PMC3035417

[9] Beltrame M, Tollervey D. Base pairing between U3 and the pre-ribosomal RNA is required for 18S rRNA synthesis. Embo J. 1995;14(17):4350–4356. 7556076 10.1002/j.1460-2075.1995.tb00109.x PMC394519

[10] Pontvianne F, Matia I, Douet J, et al. Characterization of AtNUC-L1 AtNUC - L1 reveals a central role of nucleolin in nucleolus organization and silencing of AtNUC-L2 AtNUC - L2 gene in arabidopsis. Mol Biol Cell. 2007;18(2):369–379. 17108323 10.1091/mbc.E06-08-0751 PMC1783796

[11] Zakrzewska-Placzek M, Souret FF, Sobczyk GJ, et al. Arabidopsis thaliana XRN2 is required for primary cleavage in the pre-ribosomal RNA. Nucleic Acids Res. 2010;38(13):4487–4502. 20338880 10.1093/nar/gkq172 PMC2910052

[12] Maekawa S, Ishida T, Yanagisawa S. Reduced expression of APUM24, encoding a novel rRNA processing factor, induces sugar-dependent nucleolar stress and altered sugar responses in Arabidopsis thaliana. Plant Cell. 2018;30(1):209–227. 29242314 10.1105/tpc.17.00778 PMC5810573

[13] Missbach S, Weis BL, Martin R, et al. 40S ribosome biogenesis co-factors are essential for gametophyte and embryo development. PLoS One. 2013;8(1):e54084. 23382868 10.1371/journal.pone.0054084 PMC3559688

[14] Weis BL, Palm D, Missbach S, et al. atBRX1-1 and atBRX1-2 are involved in an alternative rRNA processing pathway in Arabidopsis thaliana. RNA. 2015b;21(3):415–425. 25605960 10.1261/rna.047563.114 PMC4338337

[15] Klinge S, Woolford JL Jr. Ribosome assembly coming into focus. Nat Rev Mol Cell Biol. 2019;20(2):116–131. 30467428 10.1038/s41580-018-0078-y PMC7725133

[16] Weis BL, Kovacevic J, Missbach S, et al. Plant-Specific features of ribosome biogenesis. Trends Plant Sci; 2015a. 10.1016/j.tplants.2015.07.003 26459664

[17] Choque E, Schneider C, Gadal O, et al. Turnover of aberrant pre-40S pre-ribosomal particles is initiated by a novel endonucleolytic decay pathway. Nucleic Acids Res. 2018;46(9):4699–4714. 29481617 10.1093/nar/gky116 PMC5961177

[18] Palm D, Streit D, Shanmugam T, et al. Plant-specific ribosome biogenesis factors in Arabidopsis thaliana with essential function in rRNA processing. Nucleic Acids Res. 2019;47(4):1880–1895. 30576513 10.1093/nar/gky1261 PMC6393314

[19] Boulon S, Westman BJ, Hutten S, et al. The nucleolus under stress. Mol Cell. 2010;40(2):216–227. 20965417 10.1016/j.molcel.2010.09.024 PMC2987465

[20] Hayashi K, Matsunaga S. Heat and chilling stress induce nucleolus morphological changes. J Plant Res. 2019;132(3):395–403. 30847615 10.1007/s10265-019-01096-9 PMC7198650

[21] Kalinina NO, Makarova S, Makhotenko A, et al. The multiple functions of the nucleolus in plant development, disease and stress responses. Front Plant Sci. 2018;9:132. 29479362 10.3389/fpls.2018.00132 PMC5811523

[22] Stepinski D. Functional ultrastructure of the plant nucleolus. Protoplasma. 2014;251(6):1285–1306. 24756369 10.1007/s00709-014-0648-6 PMC4209244

[23] Ghoshal K, Jacob ST. Heat shock inhibits pre-rRNA processing at the primary site in vitro and alters the activity of some rRNA binding proteins. J Cell Biochem. 1996;62(4):506–515. 8891896 10.1002/(sici)1097-4644(19960915)62:4<506::aid-jcb8>3.0.co;2-q

[24] Coccia M, Rossi A, Riccio A, et al. Human NF-kappaB repressing factor acts as a stress-regulated switch for ribosomal RNA processing and nucleolar homeostasis surveillance. Proc Natl Acad Sci U S A. 2017;114(5):1045–1050. 28096332 10.1073/pnas.1616112114 PMC5293105

[25] Matsumura Y, Ohbayashi I, Takahashi H, et al. A genetic link between epigenetic repressor AS1-AS2 and a putative small subunit processome in leaf polarity establishment of Arabidopsis. Biol Open. 2016;5(7):942–954. 27334696 10.1242/bio.019109 PMC4958277

[26] Shinohara N, Ohbayashi I, Sugiyama M. Involvement of rRNA biosynthesis in the regulation of CUC1 gene expression and pre-meristematic cell mound formation during shoot regeneration. Front Plant Sci. 2014;5:159. 24808900 10.3389/fpls.2014.00159 PMC4009429

[27] Hang R, Wang Z, Deng X, et al. Ribosomal RNA biogenesis and its response to chilling stress in oryza sativa. Plant Physiol. 2018;177(1):381–397. 29555785 10.1104/pp.17.01714 PMC5933117

[28] Saez-Vasquez J, Medina FJ. The plant nucleolus. In: Kader J-C, Delseny M, editors. Botanical research: incorporating advances in plant pathology. Vol. 47. San Diego: Elsevier Academic Press Inc; 2008. p. 1–46.

[29] Falahati H, Pelham-Webb B, Blythe S, et al. Nucleation by rRNA dictates the precision of nucleolus assembly. Curr Biol. 2016;26(3):277–285. 26776729 10.1016/j.cub.2015.11.065 PMC5866055

[30] Earley KW, Pontvianne F, Wierzbicki AT, et al. Mechanisms of HDA6-mediated rRNA gene silencing: suppression of intergenic Pol II transcription and differential effects on maintenance versus siRNA-directed cytosine methylation. Genes Dev. 2010;24(11):1119–1132. 20516197 10.1101/gad.1914110 PMC2878650

[31] Pontvianne F, Abou-Ellail M, Douet J, et al. Nucleolin is required for DNA methylation state and the expression of rRNA gene variants in Arabidopsis thaliana. PLoS Genet. 2010;6(11):e1001225. 21124873 10.1371/journal.pgen.1001225 PMC2991258

[32] Sims J, Sestini G, Elgert C, et al. Sequencing of the Arabidopsis NOR2 reveals its distinct organization and tissue-specific rRNA ribosomal variants. Nat Commun. 2021;12(1):387. 33452254 10.1038/s41467-020-20728-6 PMC7810690

[33] Comella P, Pontvianne F, Lahmy S, et al. Characterization of a ribonuclease III-like protein required for cleavage of the pre-rRNA in the 3’ETS in Arabidopsis. Nucleic Acids Res. 2008;36(4):1163–1175. 18158302 10.1093/nar/gkm1130 PMC2275086

[34] Gallagher JE, Dunbar DA, Granneman S, et al. RNA polymerase I transcription and pre-rRNA processing are linked by specific SSU processome components. Genes Dev. 2004;18(20):2506–2517. 15489292 10.1101/gad.1226604 PMC529538

[35] Weis BL, Missbach S, Marzi J, et al. The 60S associated ribosome biogenesis factor LSG1-2 is required for 40S maturation in Arabidopsis thaliana. Plant J. 2014;80(6):1043–1056. 25319368 10.1111/tpj.12703

[36] Ginisty H, Amalric F, Bouvet P. Nucleolin functions in the first step of ribosomal RNA processing. Embo J. 1998;17(5):1476–1486. 9482744 10.1093/emboj/17.5.1476 PMC1170495

[37] Kojima H, Suzuki T, Kato T, et al. Sugar-inducible expression of the nucleolin-1 gene of Arabidopsis thaliana and its role in ribosome synthesis, growth and development. Plant J. 2007;49(6):1053–1063. 17286797 10.1111/j.1365-313X.2006.03016.x

[38] Kondo K, Kowalski LR, Inouye M. Cold shock induction of yeast NSR1 protein and its role in pre-rRNA processing. J Biol Chem. 1992;267(23):16259–16265. 1644812

[39] Mustroph A, Juntawong P, Bailey-Serres J. Isolation of plant polysomal mRNA by differential centrifugation and ribosome immunopurification methods. Methods Mol Biol. 2009;553:109–126. 19588103 10.1007/978-1-60327-563-7_6

[40] Raska I, Koberna K, Malinsky J, et al. The nucleolus and transcription of ribosomal genes. Biol Cell. 2004;96(8):579–594. 15519693 10.1016/j.biolcel.2004.04.015

[41] Montacie C, Durut N, Opsomer A, et al. Nucleolar proteome analysis and proteasomal activity assays reveal a link between nucleolus and 26S proteasome in A. thaliana. Front Plant Sci. 2017;8:1815. 29104584 10.3389/fpls.2017.01815 PMC5655116

[42] Pontvianne F, Carpentier MC, Durut N, et al. Identification of nucleolus-associated chromatin domains reveals a role for the nucleolus in 3D organization of the a. thaliana genome. Cell Rep. 2016;16(6):1574–1587. 27477271 10.1016/j.celrep.2016.07.016 PMC5279810

[43] Tsekrekou M, Stratigi K, Chatzinikolaou G. The nucleolus: in genome maintenance and repair. Int J Mol Sci. 2017;18(7):1411. 10.3390/ijms18071411 PMC5535903 28671574

[44] Huang M, Li H, Zhang L, et al. Plant 45S rDNA clusters are fragile sites and their instability is associated with epigenetic alterations. PLoS One. 2012;7(4):e35139. 22509394 10.1371/journal.pone.0035139 PMC3324429

[45] Raje HS, Lieux ME, DiMario PJ. R1 retrotransposons in the nucleolar organizers of Drosophila melanogaster are transcribed by RNA polymerase I upon heat shock. Transcription. 2018;9(5):273–285. 30063880 10.1080/21541264.2018.1506682 PMC6150621

[46] Abou-Ellail M, Cooke R, Saez-Vasquez J. Variations in a team: major and minor variants of Arabidopsis thaliana rDNA genes. Nucleus. 2011;2:294–299. 21941113 10.4161/nucl.2.4.16561

[47] Chandrasekhara C, Mohannath G, Blevins T, et al. Chromosome-specific NOR inactivation explains selective rRNA gene silencing and dosage control in Arabidopsis. Genes Dev. 2016;30(2):177–190. 26744421 10.1101/gad.273755.115 PMC4719308

[48] Picart-Picolo A, Picart C, Picault N, et al. Nucleolus-Associated chromatin domains are maintained under heat stress, despite nucleolar reorganization in Arabidopsis thaliana. J Plant Res. 2020;133:463–470. 32372397 10.1007/s10265-020-01201-3

[49] Oakes M, Aris JP, Brockenbrough JS, et al. Mutational analysis of the structure and localization of the nucleolus in the yeast *Saccharomyces cerevisiae*. J Cell Biol. 1998;143(1):23–34. 9763418 10.1083/jcb.143.1.23 PMC2132813

[50] Fleischmann J, Rocha MA, Hauser PV. RNA polymerase II is involved in 18S and 25S ribosomal RNA transcription, in *Candida albicans*. bioRxiv. 2019;510156.

[51] Doelling JH, Gaudino RJ, Pikaard CS. Functional analysis of Arabidopsis thaliana rRNA gene and spacer promoters in vivo and by transient expression. Proc Natl Acad Sci U S A. 1993;90(16):7528–7532. 8356050 10.1073/pnas.90.16.7528 PMC47175

[52] Micol-Ponce R, Sarmiento-Manus R, Ruiz-Bayon A, et al. Arabidopsis RIBOSOMAL RNA PROCESSING7 is required for 18S rRNA maturation. Plant Cell. 2018;30(11):2855–2872. 30361235 10.1105/tpc.18.00245 PMC6305980

[53] Shanmugam T, Streit D, Schroll F, et al. Dynamics and thermal sensitivity of ribosomal RNA rRNA maturation paths in plants. J Exp Bot. 2021. DOI:10.1093/jxb/erab434 34591082

[54] Lange H, Sement FM, Gagliardi D. MTR4, a putative RNA helicase and exosome co-factor, is required for proper rRNA biogenesis and development in Arabidopsis thaliana. Plant J. 2011;68(1):51–63. 21682783 10.1111/j.1365-313X.2011.04675.x

[55] Kumakura N, Otsuki H, Tsuzuki M, et al. Arabidopsis AtRRP44A is the functional homolog of Rrp44/Dis3, an exosome component, is essential for viability and is required for RNA processing and degradation. PLoS One. 2013;8(11):e79219. 24244451 10.1371/journal.pone.0079219 PMC3820695

[56] Lange H, Holec S, Cognat V, et al. Degradation of a polyadenylated rRNA maturation by-product involves one of the three RRP6-Like proteins in arabidopsis thaliana. Mol Cell Biol. 2008;28(9):3038–3044. 18285452 10.1128/MCB.02064-07 PMC2293077

[57] Sikorska N, Zuber H, Gobert A, et al. RNA degradation by the plant RNA exosome involves both phosphorolytic and hydrolytic activities. Nat Commun. 2017;8(1):2162. 29255150 10.1038/s41467-017-02066-2 PMC5735172

[58] Sikorski PJ, Zuber H, Philippe L, et al. Distinct 18S rRNA precursors are targets of the exosome complex, the exoribonuclease RRP6L2 and the terminal nucleotidyltransferase TRL in Arabidopsis thaliana. Plant J. 2015;83(6):991–1004. 26216451 10.1111/tpj.12943

[59] Azevedo-Favory J, Gaspin C, Ayadi L, et al. Mapping rRNA 2’-O-methylations and identification of C/D snoRNAs in Arabidopsis thaliana plants. RNA Biol. 2021;18:1760–1777. 33596769 10.1080/15476286.2020.1869892 PMC8583080

[60] Durut N, Abou-Ellail M, Pontvianne F, et al. A duplicated NUCLEOLIN gene with antagonistic activity is required for chromatin organization of silent 45S rDNA in Arabidopsis. Plant Cell. 2014;26(3):1330–1344. 24668745 10.1105/tpc.114.123893 PMC4001387

[61] Salih KJ, Duncan O, Li L, et al. The composition and turnover of the Arabidopsis thaliana 80S cytosolic ribosome. Biochem J. 2020;477(16):3019–3032. 32744327 10.1042/BCJ20200385 PMC7452503

[62] Kazibwe Z, Liu AY, MacIntosh GC, et al. The ins and outs of autophagic ribosome turnover. Cells. 2019;8. 10.3390/cells8121603 PMC6952998 31835634

[63] Merret R, Carpentier MC, Favory JJ, et al. Heat shock protein HSP101 affects the release of ribosomal protein mRNAs for recovery after heat shock. Plant Physiol. 2017;174(2):1216–1225. 28381501 10.1104/pp.17.00269 PMC5462041

[64] Martinez-Seidel F, Beine-Golovchuk O, Hsieh YC, et al. Systematic review of plant ribosome heterogeneity and specialization. Front Plant Sci. 2020;11:948. 32670337 10.3389/fpls.2020.00948 PMC7332886

[65] Testillano PS, Gonzalez-Melendi P, Ahmadian P, et al. The methylation-acetylation method: an ultrastructural cytochemistry for nucleic acids compatible with immunogold studies. J Struct Biol. 1995;114(2):123–139. 7542017 10.1006/jsbi.1995.1012

