# Supplementary material for: Upon heat stress processing of ribosomal RNA precursors into mature rRNAs is compromised after cleavage at primary P site in Arabidopsis thaliana
Source: RNA Biol. 2022 May 6;19(1):719–34. doi: 10.1080/15476286.2022.2071517 (PMC9090299; doi:10.1080/15476286.2022.2071517)
Supplement: Supplemental Material [file KRNB_A_2071517_SM7003.zip › Supplemental Figures and Information_RNABiol_revised2.pdf]

Supplemental Figures

Figure S1. ***Arabidopsis thaliana* growth phenotype under and after heat stress. A)** Plants treated at 37°C for 2h to 55h (Heat stress) or **B)** treated at 37°C for 2h to 24h (Heat stress) and returned at 22°C for 5 to 31h (recovery 22°C). Control plant seedlings before treatments (22°C) are shown in A and B.

Figure S2. Transmission Electron Microscopy (TEM) pictures. 116 pictures of nucleolus morphology in Arabidopsis seedlings maintained at 22°C, treated at 37°C for 5h, 8h and 24h, and after recovery at 22°C for 24h.

Figure S3. Northern Blots analysis of total RNAs from Col-0, *nuc1-2*, nuc2-2, *rtl2* and *rh10-1* lines at 22°C and 37°C. Two independent membranes were hybridized with p5, p23, p18S, p3 (A) or with p6, p43, p18S and p4 (B). Both were hybridized withp5S, pU3 and pU6 (A and B). Panels p18S+p3 and p18S+p4 were hybridized with p18S, before p3 or p4. The p18S specific signals are shown in brackets. A relative similar amount of mature rRNAs 18S is detected at 22°C and 37°C while transcripts that might correspond to truncated 18S rRNAs accumulate at 37°C (odd lanes, red asterisks).pre-rRNAs detected with p3, p4, p5, p6, p23 and p43 are labelled accordingly to previous reports using identical probes. Pre-rRNAs detected upon heat stress are red labelled: arrows, and arrowheads. p5S, pU3 and pU6 probes detect respectively 5S rRNA, small nucleolar U3 (snoRNA U3) and small nuclear U6 (snRNA U6) RNAs. Quality and similar amount of RNAs for each sample was verified by Gelred staining. Black asterisk in the stained gel indicates a sporadic and unknown RNA species. Col-0 images (lanes 1 and 2) in A and B are used in main Figure 3B while Col-0 and nuc1-2 (lanes 1-4) images in A are used in main figure 6C.

Figure S4. **Kinetics of pre-rRNA processing throughout heat stress and recovery conditions.** Northern blot analyses of total RNAs from seedlings heat treated at 37°C for 6h, 12h, 18h,24h and 30h (lanes 1-5), and treated 24h at 37°C and then returned to 22°C conditions for 6h, 12h, 24h and 50h (lanes 6-9). Pre-rRNAs detected upon heat stress are red labelled indicated. Quality and similar amount of RNA for each sample was verified by Gel Red staining and hybridization with p5S to detect 5S rRNA.

Figure S5. Northern blot analysis of total RNAs from non-treated (lane 1), heat treated at 37°C, 24h (lane 2) or recovered R22°C-24h (lane 3) seedlings using p43 and p5S DIG-labelled probes. rRNA hybridization signals were detected with anti-digoxigenin (Supplemental Information 4). After dehybridization of p43, the membrane was incubated with p5S probe. Quality and similar amount of RNAs for each sample were also verified by Gel red staining.

Figure S6. NUC1 and U3 snoRNP expression in response to heat stress. A) Western blot analysis of total protein extracts from Col-0 (lane 1) and *nuc1-2* (lane 2) plants with α-NUC1 antibodies. **B)** Upper panel, Western blot analysis of total protein extracts from non-treated (lane 1) and heat treated (37°C)

for 24h (lane 2) seedlings with α-FIB antibodies. Actin is used as loading control. Lower panel, FIB1 (At5g52470) and FIB2 (At4g25630) transcript levels in non-treated (22°C) and heat treated (37°C, 24h) plants. **C)** Primer extension analysis performed on total RNAs from Arabidopsis plants maintained at 22°C or treated for 24h at 37°C (same as lanes 1 and 8, Figure 4) and using primer oU3 to detect U3 snoRNA. Mock control reaction (lane 1) using yeast tRNA.

Figure S7. **Ribosome profiles and LC-MS/MS analysis. A)** Extracts from non-treated (22°C) and heat treated (37°C for 6h) seedlings were fractionated through 15-60% sucrose gradients. The percentage of the full scale of absorbance was monitored at 254 nm. Peaks corresponding to 40S and 60S-80S ribosomal subunits/monosomes are indicated. The ratios of the 60-80S over the 40S and fraction analysed by LS-MS/MS are indicated **B)** Table shows percent of spectra associated to ribosomal proteins RPL, RPS and RPP in relation to all RP proteins identified in fraction 8-11 from samples at 22°C and fractions 9-12 from 37°C, 6h samples. The ratio RPS/RPL for each fraction and condition was calculated.

Figure S8. **Compartmental modelling of the 45S pre-rRNA processing**. **A)** Flow-diagram representing the pre-rRNA intermediates whose dynamics are modelled according to the 45S production rate (B45), the processing rate of each *pre-rRNA* (k_{45S}, k_{35S} , $k_{P\text{-}A3}$) and proportion of the 35S that is degraded into P-A3 (a). **B)**. Estimates of the model parameters obtained by fitting the model to the temporal variations in *pre-rNA intermediates* densities observed during heat stress (red) and recovery (green) periods using the p43 (circles) and p23 (triangle) probes. All parameter estimates are measured per hour, but the probability a. Supplementary methods and raw data used to generate the compartmental modelling are provided in Table S4 and Supplementary Information 2.

Figure S9. pre-RNA processing in *rrp7-1* in response to heat-stress. Northern Blot analysis of total RNAs from Arabidopsis WT and *rrp7-1* seedlings in non-treated (22°C, lane 1 and 3) and heat-treated (37°C for 24h, lanes 2 and 4) using probe p23. Pre-rRNAs detected specifically in the heat stressed plant samples are indicated in red: 45S, arrows, and arrowheads. Similar amounts of RNAs in each sample were verified by Gel Red staining and hybridization with p5S to detect 5S rRNA.

Figure S10. **Expression of nucleases and related activities required for pre-rRNA processing. A)** Transcript levels in non-treated (22°C) and heat treated (37°C for 24h) plants for endonucleases LAS1-like (At5g12220), MRP1/POP1 (At2g47300) and MRP1/POP4 (At2g43190), UTP24 (At2g46230), NOB1 (At5g41190), RCL1 (At5g22100), RTL2 (At3g20420), exonucleases XRN2 (At5g42540), XRN3 (At1g75660), NOL12 (At1g11240), DIS3/RRP44A (At2g17510), RRP6L2 (At5g35910), RRP41 (At3g61620) and TRL (At5g53770) and MTR4 (At1g59760). **B)** Representation of 45 pre-rRNA transcripts shows cleavages sites in the 5'ETS, ITS1, ITS2 and 3'ETS in Arabidopsis. rRNA site targeted by U3 snoRNP complex and endonucleases Utp4/UTP24, Nob1/NOB1, Rcl1, MRP, Las1/LAS1L and Rnt1/RTL2 are indicated. Numbers in green correspond to site positions in *Arabidopsis thaliana* 45S rRNA mapped in this work. p values (student's t test) were calculated using RStudio (R version 4.0.4). **C)** Heat map of 242 ribosomal proteins and 99 ribosome biogenesis factors

from 15 day-old *in vitro* grown plants, heat treated (37°C for 24h) and non-heat treated (mock, 22°C). The color key, histogram and values are shown. Down and up regulated genes are in blue and red respectively.

Supplementary Information 1: 45S rDNA sequence (8086 nt)

AGGGGGGTGGGTGTTGAGGGAGTCTGGGCAGTCCGTGGGGAACCCCCTTTTTCGGTTCGGACT
TGGGTAGCGATCGAGGGATGGTATCGGATATCGGCACGAGGAATGACCGACCGTCCGGCCGCC
GGGATTTTCGCCGGAAAACTTTTCCGGGCACTTTTCCGGCGATCGGTTTTGTTGCCTTTTTCCGA
GTTTTCTCAGCAGTTCTCGGACAAAAACTGCTGAATCGTCGAGGAGAATGGGCTTGCTTGCGTGG
GCTGCCATTAGTTCTTCGAGGCGTTAGGGTGGCGGCGGTATAAAAGTGTCGGAGTTTTTTCAGCA
GTTCTCGGACAAAAATTGCTGAGTGGCCGAGAAGAATGGGCGTGTCATGCGTGGGCTGACATGG
ATTCTTCGAGGCCTAGGGGTGGCGGTATATAACTTGTTCGCATGATATTACCGAGATGTCCCCAC
GGGCATCTTTTCACCTCGTCGCCGAAGAGAATGGGCGTGTCATGGCATGGGCTGACATGGATTC
TCCTAGGCCGTTTGGGTGGCGGTATAGTCGTCTTGCGCACGAAATACCGAGATGTCCCCATGGG
CATCGATTCCACCCGCCTAGGTTGGATGGGCGTGCTTCGTCGGAAAGCATGGATCCGCCTAGGC
TGTCCCGAGTGTGAGCGAGGTGTGAGTGTCGCCCATGGGCATCGACACCTTGCGGCTAGGAACT
GGAACGAGACGGGTGGCAAAGATTTCGAGTAGCACTTCATACTACCGTGGGTTTTTTAAACCTTC
CGAGTTTTGTTGATGTTATTCCGAGAATTAGCAAACCGTAACGAAGATGTTCTTGGCAACCATCTT
TTGATGGGAGTCCGGCTGTTCGATAGCCGGCCAAGGGTGATGAACGAAATGTGAACCCTTGTCT
CGCCTAGGTTGGATGGGCGTGCTTCGTTGGAAAGCATGGATCCGCCTAGGCTGTCCCGAGTGTG
AGCGAGGTGTGAGTGTCGCCCATGGGCATCGACACCTTGCGGCTAGGAACTGGAACGAGACGG
GTAGCAAAGATTTCGAGTAGCACTTCATACTACCGTGGGTTTTTTAAACCTTCCTAGTTTTGTTGAT
GTTATTCCGAGAATTAGCAAACCGTAACGAAGATGTTCTTGGCAACCATCTTTTGATGGGAGTCC
GGCTGTTCGAAAGCCGGCCAAGGGTGATGAACGAAATGTGAACCCTTGTCTCGCCTAGGTTGGA
TGGGCGTGCTTCGTTGGAAAGCATGGATCCGCCTAGGCTGTCCCGAAGTATCTCGCGCTTGTAC
GGCTTTGGCTCGGATTCGTCCGTCTTCTTTCTTCTTAGCCGAGTACTTCGGTAGATTAGTTGGAAC
GATTGATGATTTTGAGTTAATTGAACGTTCGGCGTATGAGTGGTGATCGGATAGCTAGTGTTCGTA
GGCTCCATGCTCGCGCATCGAACTACCTACCACCTATCCTTCTCAGTTAATTCACGGGCGATGTT
ACGCTCGATGATGAGTTCCGGGGCCTGTGTTTCGTACCTAATTTGAAGGAATTGTTGAGTTTGGT
TTACACCTTTGCCCGCGGCTTCTCCTTCGTGGGGAAGTCGTGGGCTCAAACATCGGCGCTTGTT
CACCTCTCGTCATCGCATTTGTTGCCTTGCTCGCATTGGTGAATGAGTTGCGGGTTGAAATCTCG
GATGCGGAAAAGTTGTCGACGGTGACTCGAAGTGATTCAGTCCCGCCAAAGCTCATCCGTCCTT
CGGGCAAAAGATGACGGTCAAGACCTCGTCCTTTCTCTCTTTCCATTGCGTTTGAGAGGATGTGG
CGGGGAATTGCCGTGATCGATGAATGCTACCTGGTTGATCCTGCCAGTAGTCATATGCTTGTCTC
AAAGATTAAGCCATGCATGTGTAAGTATGAACGAATTCAGACTGTGAAACTGCGAATGGCTCATTA
AATCAGTTATAGTTTGTTTGATGGTAACTACTACTCGGATAACCGTAGTAATTCTAGAGCTAATAC
GTGCAACAAACCCCGACTTATGGAAGGGACGCATTTATTAGATAAAAGGTCGACGCGGGCTCTG
GCTTGCTCTGATGATTCATGATAACTCGACGGATCGCATGGCCTCTGTGCTGGCGACGCATCATT
CAAATTTCTGCCCTATCAACTTTCGATGGTAGGATAGTGGCCTACCATGGTGGTAACGGGTGACG
GAGAATTAGGGTTCGATTCCGGAGAGGGAGCCTGAGAAACGGCTACCACATCCAAGGAAGGCAG
CAGGCGCGCAAATTACCCAATCCTGACACGGGGAGGTAGTGACAATAAATAACAATACCGGGCT
CTTTCGAGTCTGGTAATTGGAATGAGTACAATCTAAATCCCTTAACGAGGATCCATTGGAGGGCA
AGTCTGGTGCCAGCAGCCGCGGTAATTCCAGCTCCAATAGCGTATATTTAAGTTGTTGCAGTTAA
AAAGCTCGTAGTTGAACCTTGGGATGGGTCGGCCGGTCCGCCTTTGGTGTGCATTGGTCGGCTT
GTCCCTTCGGTCGGCGATACGCTCCTGGTCTTAATTGGCCGGGTCGTGCCTCCGGCGCTGTTAC
TTTGAAGAAATTAGAGTGCTCAAAGCAAGCCTACGCTCTGGATACATTAGCATGGGATAACATCAT
AGGATTTCGATCCTATTGTGTTGGCTTCGGGATCGGAGTAATGATTAACAGGGACAGTCGGGGG
CATTCGTATTTCATAGTCAGAGGTGAAATTCTTGGATTTATGAAAGACGAACAACTGCGAAAGCAT
TTGCCAAGGATGTTTTCATTAATCAAGAACGAAAGTTGGGGGCTCGAAGACGATCAGATACCGTC
CTAGTCTCAACCATAAACGATGCCGACCAGGGATCAGCGGATGTTGCTTATAGGACTCCGCTGG
CACCTTATGAGAAATCAAAGTTTTTGGGTTCCGGGGGGAGTATGGTCGCAAGGCTGAAACTTAAA
GGAATTGACGGAAGGGCACCACCAGGAGTGGAGCCTGCGGCTTAATTTGACTCAACACGGGGAA
ACTTACCAGGTCCAGACATAGTAAGGATTGACAGACTGAGAGCTCTTTCTTGATTCTATGGGTGG
TGGTGCATGGCCGTTCTTAGTTGGTGGAGCGATTTGTCTGGTTAATTCCGTTAACGAACGAGACC
TCAGCCTGCTAACTAGCTACGTGGAGGCATCCCTTCACGGCCGGCTTCTTAGAGGGACTATGGC
CGTTTAGGCCAAGGAAGTTTGAGGCAATAACAGGTCTGTGATGCCCTTAGATGTTCTGGGCCGCA
CGCGCGCTACACTGATGTATTCAACGAGTTCACACCTTGCCGACAGGCCCGGGTAATCTTTGAAA
TTTCATCGTGATGGGGATAGATCATTGCAATTGTTGGTCTTCAACGAGGAATTCCTAGTAAGCGC
GAGTCATCAGCTCGCGTTGACTACGTCCCTGCCCTTTGTACACACCGCCCGTCGCTCCTACCGAT
TGAATGATCCGGTGAAGTGTTCGGATCGCGGCGACGTGGGTGGTTCGCCGCCCGCGACGTCGC
GAGAAGTCCACTAAACCTTATCATTTAGAGGAAGGAGAAGTCGTAACAAGGTTTCCGTAGGTGAA
CCTGCGGAAGGATCATTGTCGATACCTGTCCAAAACAGAACGACCCGCGAACCAAAGATCACCA
CTCTCGGTGGGCCGGTTTCTTAGCCGATTCCTTGCCCGCCGGATCCGTGGTTTCGCGTATCGGC

ATGATCGGGAGCTTTTATCTCGGTCTTGTCGTGCGCGTTGCTTCCGGATATCACAAAACCCCGGC
ACGAAAAGTGTCAAGGAACATGCAAACGAACGGCTGGCATTCGCCTCCCCGGAGACGGAGTGTG
GGCGGATGCTGTGCTGCGAACTGAAGTCTAAAACGACTCTCGGCAACGGATATCTCGGCTCTCG
CATCGATGAAGAACGTAGCGAAATGCGATACTTGGTGTGAATTGCAGAATCCCGTGAACCATCGA
GTCTTTGAACGCAAGTTGCGCCCCAAGCCTTCTGGCCGAGGGCACGTCTGCCTGGGTGTCACAA
ATCGTCGTCCCTCACCATCCTTTGCTGATGCGGGACGGAAGCTGGTCTCCCGTGTGTTACCGCA
CGCGTTGGCCTAAATCCGAGCCAAGGACGCCTGGAGCGTACCGACATGCGGTGGTGAACTTGAT
CCATTACATTTTATCGGTCGCTCTTGTCCGGAAGCTGTAGATGACCCAAAGTCCATATAGCGACC
CCAGGTCAGGCGGGATTACCCGCTGAGTTTAAGCATATCAATAAGCGGAGGAAAAGAAACTAACA
AGGATTCCCTTAGTAACGGCGAGCGAACCGGGAAGAGCCCAGCTTGAAAATCGGACGTCTTCGG
CGTTCGAATTGTAGTCTGGAGAAGCGTCCTCAGCGACGGACCGGGCCTAAGTTCCCTGGAAAGG
GGCGCCAGAGAGGGTGAGAGCCCGTCGTGCCCGGACCCTGTCGCACCACGAGGCGCTGTCTAC
GAGTCGGGTTGTTTGGGAATGCAGCCCCAATCGGGCGGTAAATTCCGTCCAAGGCTAAATACGG
GCGAGAGACCGATAGCGAACAAGTACCGCGAGGTAAAGATGAAAAGGACTTTGAAAAGAGAGTC
AAAGAGTGCTTGAAATTGTCGGGAGGGAAGCGGATGGGGGCCGGCGATGCGTCCTGGTCGGAT
GCGGAACGGAGCAATCCGGTCCGCCGATCGATTCGGGGCGTGGACCGACGCGGATTACGGTGG
CGGCCTAAGCCCGGGCTTTTGATACGCTTGTGGAGACGTCGCTGCCGTGATCGTGGTCTGCAGC
ACGCGCCTAACGGCGTGCCTCGGCATCAGCGTGCTCCGGGCGTCGGCCTGTGGGCTCCCCATT
CGACCCGTCTTGAAACACGGACCAAGGAGTCTGACATGTGTGCGAGTCAACGGGTGAGTAAACC
CGTAAGGCGCAAGGAAGCTGATTGGCGGGATCCTCGCGGGTGCACCGCCGACCGACCTTGATC
TTCTGAGAAGGGTTCGAGTGTGAGCATGCCTGTCGGGACCCGAAAGATGGTGAACTATGCCTGA
GCGGGGTAAAGCCAGAGGAAACTCTGGTGGAAGCCCGCAGCGATACTGACGTGCAAATCGTTC
GTCTGACTTGGGTATAGGGGCGAAAGACTAATCGAACCATCTAGTAGCTGGTTCCCTCCGAAGTT
TCCCTCAGGATAGCTGGAGCTCGGACGCGAGTTCTATCGGGTAAAGCCAATGATTAGAGGCATT
GGGGGCGCAACGCCTCGACCTATTCTCAAACTTTAAATAGGTAGGACGTGTCGGCTGCTTTGTTG
AGCCGTCACACGGAATCGAGAGCTCCAAGTGGGCCATTTTTGGTAAGCAGAACTGGCGATGCGG
GATGAACCGGAAGCCGGGTTACGGTGCCCAACTGCGCGCTAACCTAGAACCCACAAAGGGTGTT
GGTCGATTAAGACAGCAGGACGGTGGTCATGGAAGTCGAAATCCGCTAAGGAGTGTGTAACAAC
TCACCTGCCGAATCAACTAGCCCCGAAAATGGATGGCGCTTAAGCGCGACCTATACCCGGCCGT
CGGGGCAAGAGCCAGGCCTCGATGAGTAGGAGGGCGCGGCGGTCGCTGCAAAACCTAGGGCG
CGAGGCGCGGAGCGGCCGTCGGTGCAGATCTTGGTGGTAGTAGCAAATATTCAAATGAGAACTT
TGAAGGCCGAAGAGGGGAAAGGTTCCATGTGAACGGCACTTGCACATGGGTTAGTCGATCCTAA
GAGTCGGGGGAAACCCGTCTGATAGCGCTTAAGCGAACTTCGAAAGGGGATCCGGTTAAAATTC
CGGAACCGGGACGTGGCGGTTGACGGCAACGTTAGGGAGTCCGGAGACGTCGGCGGGGGCCT
CGGGAAGAGTTATCTTTTCTGTTTAACAGCCTGCCCACCCTGGAAACGGCTCAGCCGGAGGTAG
GGTCCAGCGGCTGGAAGAGCACCGCACGTCGCGTGGTGTCCGGTGCGCCCCCGGGCGCCCTT
GAAAATCCGGAGGACCGAGTGCCGCTCACGCCCGGTCGTACTCATAACCGCATCAGGTCTCCAA
GGTGAACAGCCTCTGGTCGATGGAACAATGTAGGCAAGGGAAGTCGGCAAAATGGATCCGTAAC
TTCGGGAAAAGGATTGGCTCTGAGGGCTGGGCTCGGGGGTCCCAGTTCCGAACCCGTCGGCTG
TCAGCGGACTGCTCGAGCTGCTTCCGCGGCGAGAGCGGGTCGCCGGCTGCCGGCCGGGGGAC
GACTGGGAACGGCTCTCTCGGGAGCTTTCCCCGGGCGTCGAACAGTCAGCTCAGAACTGGTAC
GGACAAGGGGAATCCGACTGTTTAATTAAAACAAAGCATTGCGATGGTCCCTGCGGATGCTAACG
CAATGTGATTTCTGCCCAGTGCTCTGAATGTCAAAGTGAAGAAATTCAACCAAGCGCGGGTAAAC
GGCGGGAGTAACTATGACTCTCTTAAGGTAGCCAAATGCCTCGTCATCTAATTAGTGACGCGCAT
GAATGGATTAACGAGATTCCCACTGTCCCTGTCTACTATCCAGCGAAACCACAGCCAAGGGAACG
GGCTTGGCAGAATCAGCGGGGAAAGAAGACCCTGTTGAGCTTGACTCTAGTCCGACTTTGTGAA
ATGACTTGAGAGGTGTAGGATAAGTGGGAGCTTCGGCGCAAGTGAAATACCACTACTTTTAACGT
TATTTTACTTACTCCGTGAATCGGAGGCCGGGGTACAACCCCTGTTTTTGGTCCCAAGGCTCGCT
TCGGCGGGTCGATCCGGGCGGAGGACATTGTCAGGTGGGGAGTTTGGCTGGGGCGGCACATCT
GTTAAAAGATAACGCAGGTGTCCTAAGATGAGCTCAACGAGAACAGAAATCTCGTGTGGAACAAA
AGGGTAAAAGCTCGTTTGATTCTGATTTTCAGTACGAATACGAACCGTGAAAGCGTGGCCTATCG
ATCCTTTAGACTTCGGAATTTGAAGCTAGAGGTGTCAGAAAAGTTACCACAGGGATAACTGGCTT
GTGGCAGCCAAGCGTTCATAGCGACGTTGCTTTTTGATCCTTCGATGTCGGCTCTTCCTATCATT
GTGAAGCAGAATTCACCAAGTGTTGGATTGTTCACCCACCAATAGGGAACGTGAGCTGGGTTTAG
ACCGTCGTGAGACAGGTTAGTTTTACCCTACTGATGCCCGCGTCGCGATAGTAATTCAACCTAGT
ACGAGAGGAACCGTTGATTCGCACAATTGGTCATCGCGCTTGGTTGAAAAGCCAGTGGCGCGAA
GCTACCGTGCGCTGGATTATGACTGAACGCCTCTAAGTCAGAATCCGGGCTAGAAGCGACGCAT
GCGCCCGCCGCCCGATTGCCGACCCTCAGTAGGAGCTTAGGCTCCAAAGGCACGTGTCGTTGG
CTAAGTCCGTTCGGCGGAACGGTCGTTCGGACCGCCTTGAATTATAATTACCACCGAGCGGCGG
GTAGAATCCTTTGCAGACGACTTAAATACGCGACGGGGTATTGTAAGTGGCAGAGTGGCCTTGCT

GCCACGATCCACTGAGATTCAGCCCTTTGTCGCTAAGATTCGACCCTCCCCTAAATCACTCCAAA
AAAAACAATCCCCAATTCTACACAAGTGTTTCTACACTAACAAAGCAACAGCTCCTTAACGAATTC
CCAACTTTACACGAGCTCGTCTCTCGAGGTTAAATGTTATTACTTGGTAAGATTCCGGACCTCGCC
AAGTGTTTTGAAAACCCGCAACGCTCGCAAAGGTGGATAGTGAGAATAATAAGTGAAGAGACAGA
CTTGTCCAAAACGCCCACCACGAAGGTGCATAGTGAGAAGAGTAAGTCAAGAGATAGACTTGTCC
AAAAAGAAACGGAAGAGAAAGCGTGGGGAGACGCTCACGAAGGTGCATAGTGAGAAGAGTAAGT
CAAGAGACAGACTTGTTCGAAAAGAAACAGAAGAGAATGCTTGGGGTTACACTCACGAAGGTGCA
TAGTGAGAAGAGTAAGTCAAGAGACAGACTTGTTCGAAAA

Supplementary Information 2: Compartmental model

1. Model

Let's denote $w(t)$, $x(t)$ and $y(t)$, the quantity of 45S, 35S and PA_3 at time t. The system of ordinary differential equations describing their time dynamic can be written down as follow;

$$\dot{w}(t) = B_{45} - k_{45S}w(t) \quad \textbf{Eq. 1}$$

$$\dot{x}(t) = +k_{45S}w(t) - k_{35S}x(t) \quad \textbf{Eq. 2}$$

$$\dot{y}(t) = +\alpha k_{35S}x(t) - k_{PA_3}y(t) \quad \textbf{Eq. 3}$$

where $\dot{w}(t)$, $\dot{x}(t)$ and $\dot{y}(t)$ denote time derivative of the corresponding state variables.

2. Explicit solutions of the model.

21. Solution for the time dynamic of the amount of 45S

Integrating over time equation 1 leads to

$$w(t) = C(t)e^{-k_{45S}\,t}$$

where

$$C(t) = \int B_{45}\, e^{k_{45S}\,t} + B$$

that can easily be shown to be equal to

$$C(t) = \frac{B_{45}}{k_{45S}}\, e^{k_{45S}\,t} + B \quad \text{with } A = \frac{B_{45}}{k_{45S}}$$

This allows, after the calculation of B by considering the initial condition, to write down the explicit solution for the amount of 45S:

$$w(t) = A + Be^{-k_{45S}\,t} \quad \textbf{Eq. 4}$$

with $B = w(0) - A$

22. Solution for the time dynamic of the amount of 35S

Equation 2 can be re-written using the expression of $w(t)$:

$\dot{x}(t) = f(t) - k_{35S}x(t)$ **Eq. 2'**

where $f(t) = k_{45S}\,(A + Be^{-k_{45S}\,t})$

Integrating over time equation 2' leads to

$x(t) = C(t)e^{-k_{35S}\,t}$

where

$$C(t) = \int f(t)\,e^{k_{35S}\,t} + C'$$

that is

$C(t) = A'e^{k_{35S}\,t} + Be^{(k_{35S}-k_{45S})t} + C'$ with $A' = \frac{k_{45S}}{k_{35S}}A$ and $B' = \frac{k_{45S}}{k_{35S}-k_{45S}}B$

This allows, after the calculation of C' by considering the initial conditions, to write down the explicit solution for the amount of 35S:

$x(t) = A' + B'e^{-k_{45S}\,t} + C'e^{-k_{35S}\,t}$ **Eq. 5**

with $C' = x(0) - (A' + B')$

23. Solution for the time dynamic of the amount of PA3

Equation 3 can be re-written using the expression of (t) :

$\dot{y}(t) = g(t) - k_{PA_3}y(t)$ **Eq. 3'**

where $g(t) = \alpha k_{35S}\,(A' + B'e^{-k_{45S}\,t} + C'e^{-k_{35S}\,t})$

Integrating over time equation 3' leads to

$y(t) = C(t)e^{-k_{PA3}\,t}$

where

$$C(t) = \int g(t)\,e^{k_{PA3}\,t} + D''$$

that is

$C(t) = A''e^{k_{PA3}\,t} + B''e^{(k_{PA3}-k_{45S})t} + C''e^{(k_{PA3}-k_{35S})t} + D''$

with $A'' = \frac{\alpha k_{35S}}{k_{PA3}}A'$, $B'' = \frac{\alpha k_{35S}}{k_{PA3}-k_{45S}}B'$ and $C'' = \frac{\alpha k_{35S}}{k_{PA3}-k_{35S}}C'$

This allows, after the calculation of C' by considering the initial conditions, to write down the explicit solution for the amount of PCA_3 :

$$y(t) = A'' + B''e^{-k_{45S}\,t} + C''e^{-k_{35S}\,t} + D''e^{-k_{PA3}\,t} \qquad \textbf{Eq. 6}$$

with $D'' = y(0) - (A'' + B'' + C'')$

3. Asymptotic Equilibrium

The asymptotic equilibrium of 45S, 35S and PA_3 can be found by taking time to infinity in equations 4-6. This leads to the following equilibrium levels:

$$w^* = A = \frac{B_{45}}{k_{45S}} \qquad \textbf{Eq. 7}$$

$$x^* = A' = \frac{k_{45S}}{k_{35S}} A = \frac{B_{45}}{k_{35S}} \qquad \textbf{Eq. 8}$$

$$y^* = A'' = \frac{\alpha k_{35S}}{k_{PA3}} A' = \frac{\alpha B_{45}}{k_{PA3}} \qquad \textbf{Eq. 9}$$

Supplementary Information 3: LC-MS/MS

Protein purification

The 500µl sucrose fractions (8-11 from 22°C samples and 9-12 from 37°C samples, Figure S4) in 0.2M Tris pH 9.0, 0.2 M KCl, 0.025 M EGTA, 0.35 M MgCl2, 5 mM DTT, 50 µg/ml cycloheximide, 50 µg/ml chloramphenicol and ~30%.sucrose were cleaned using a Filter-Aided Sample Preparation (FASP) adapted from Wiśniewski et al. (Wisniewski et al. 2009) to remove sucrose and buffer. Five fmol of Bovine Serum Albumin (BioRad, Des Plaines, USA) standard protein was added to each fraction as a positive control of the sample preparation.

Half of the sucrose fractions were mixed with 200 µl of 8 M urea 0.1 M Tris HCl pH 8.5 buffer in Microcon-10 kDa filter units (Merck, Darmstadt, Germany). The device was centrifuged at 14,000 g for 40 min with slight heating at 28°C to favour sucrose release. All following centrifugation steps were performed applying the same conditions. The same procedure was repeated for the second half of the fractions. For high sucrose fractions, samples were further diluted with 100 µl of water prior to extra centrifugation steps. Samples were incubated with 100 µl of 10 mM dithiothreitol in urea buffer at 37°C for 30 min. Subsequently, 100 µl of 0.05 M iodoacetamide in urea buffer were added for 20 min in dark at room temperature followed by centrifugation. The resulting concentrate was diluted with 100 µl resuspension buffer (Promega, Madison, USA) and concentrated. This step was repeated twice, and the concentrate was subjected to proteolytic digestion for one hour at 70°C with 100 µl rapid Trypsin/LysC (Promega, Madison, USA) added in a 1/10 ratio. Trypsin digests were collected by centrifugation, and the filter device was rinsed twice with 70 µl 0.5 M NaCl and centrifuged. The combined peptidic filtrates were acidified with 4 µl of pure formic acid and desalted on 5 µl C18 SPE cartridges on a Bravo AssayMap (Agilent Technologies, Santa Clara, USA). Two third of the total peptides extracts were injected on a LC-MS/MS coupling.

Liquid Chromatography-Tandem Mass Spectrometry (LC-MS/MS) Analyses

LC-MS/MS analyses of peptide extracts were performed on a NanoAcquity LC-system (Waters, Milford, MA, USA) coupled to a Q-Orbitrap (Q-Exactive Plus from Thermo Fisher Scientific, Waltham, MA, USA) mass spectrometer equipped with a nanoelectrospray ion source. Mobile phase A (99.9% water and 0.1% FA) and mobile phase B (99.9% acetonitrile and 0.1%FA) were delivered at 400 nL/min. Samples were loaded into a Symmetry C18 precolumn (0.18 x 20 mm, 5 µm particles size, Waters) over 3 minutes in 1% buffer B at a flow rate of 5 µL/min. This step was followed by reverse-phase separation at a flow rate of 400 nL/min using an ACQUITY UPLC® BEH130 C18 separation column (250 mm x 75 µm id, 1.7 µm particles size, Waters). Peptide mixtures were eluted using a gradient from 1% to 35% B in 79 minutes, from 35% B to 90% B in 1 minute, maintained at 90% B for 5 minutes and the column was reconditioned at 1% B for 20 minutes.

The Q-Orbitrap instrument was operated in data dependent acquisition mode by automatically switching between full MS and consecutive MS/MS acquisitions. Survey full scan MS spectra (mass range 300-1800) were acquired with a resolution of 70,000 at 200 m/z with an automatic gain control (AGC) fixed at 3×10^6 ions and a maximum injection time set at 50 ms. The ten most intense peptide ions in each survey scan with a charge state >= 2 were selected for MS/MS fragmentation. MS/MS scans were performed at 17,500 resolution at 200 m/z with a fixed first mass at 100 m/z, AGC was fixed at 1×10^5 and the maximum injection time was set to 100 ms. Peptides were fragmented by higher-energy collisional dissociation (HCD) with a normalised collision energy set to 27. Peaks selected for fragmentation were automatically put on a dynamic exclusion list for 60 s and peptide match selection was turned on. MS data were saved in .raw file format (Thermo Fisher Scientific) using XCalibur.

LC-MS/MS data interpretation

Raw files were converted to .mgf peaklists using MsConvert (using MSAngel) and were submitted to Mascot database searches (version 2.6.2, MatrixScience, London, UK) against an *Arabidopsis thaliana* protein sequences database downloaded from *The Arabidopsis Information Resource TAIR* site (TAIR10 version gene model), common contaminants and decoy sequences were added. The concatenated database contains 2 x 27 534 protein entries. Spectra were searched with a mass tolerance of 15 ppm in MS mode and 0.07 Da in MS/MS mode. One trypsin missed cleavage was tolerated. Carbamidomethylation of cysteine residues was set as a fixed modification. Oxidation of methionine residues and acetylation of protein n-termini were set as variable modifications. Identification results were imported into Proline software (http://proline.profiproteomics.fr/) for validation. Peptide Spectrum Matches (PSM) with pretty rank equal to one were retained. False Discovery Rate was then optimized to be below 1% at PSM level using Mascot E-value and below 1% at Protein Level using Mascot score and more than one specific peptide. Protein abundances of RPS, RPL and RPP ribosomal proteins were estimated using weighted spectral counts (Bouyssie et al. 2020).

Supplementary Information 4: Northern blot with DIG probes

For RNA gel blot analysis, three µg of total RNA were loaded into 1% (w/v) agarose gel in 1X TT buffer (50X TT buffer: 1.5M Tricine, 1.5M Triethanolamine) containing 0.5% formaldehyde and 5 µl of GelRed/100 ml. RNA samples (lyophilized) were resuspended in 10 µl of formamide and 10 µl of loading buffer (2.1X TT buffer, 1 mM EDTA; 0.04% Blue de Bromophenol) supplemented with 2.5% formaldehyde; then denaturated at 70°C for 5 min. The gel was run for 3 to 4 h at 100 V in 1X TT buffer and RNA visualized using a Vilber Smart Imaging system, transferred (capillary in 10X SSC) and UV cross-linked (Stratalinker) onto a Hybond N+ nylon membrane (Roche).

RNA Hybridization was carried out as previously described (Micol-Ponce et al. 2018; Micol-Ponce et al. 2020) using Digoxigenin-labeled probes Briefly, pre-hybridization was performed in 1X PerfectHyb^{TM} Plus Hybridization Buffer (Roche) at 42°C for 3h. Then DIG-oligo probes were added to final concentration of 0.015 µM and incubated over-night at 42°C. Membranes were washed first with 2X SSC, 0.1% SDS for 30 min at 50°C and second with 0.5X SSC, 0.1% SDS for 30 min at 50°C.

Detection of DIG probes was performed following Roche's instructions. Membranes were washed maleic acid buffer, blocked with blocking reagent and hybridized with anti-digoxigenin-AP 1:15,000 (Roche) over night at 4°C. Membranes were washed and hybridization signals detected with CDP-*Star* solution and visualized using Fusion-Solo S software (Vilber Smart Imaging).

Probes p6, p23, p43, p5S; p5.8S and p18S were 5′ end Digoxigenin-labeled oligonucleotides, and were synthetized by (Roche). The sequences of the primers and oligonucleotides used to obtain all probes are described in Supplemental Table S1.

Supplementary Information 5: RNAseq

RNA sequencing was performed by the platform MGX BioCampus Montpellier, France using total RNAs from 15 days-old *in vitro* grown plants heat treated (37°C for 24h) and non-heat treated (mock, 22°C) and Illumina Hiseq 2000 Sequencing System to generate 50 bp short reads. Reads were mapped to the *Arabidopsis thaliana* genome (TAIR10) using Tophat v. 2.0.0 and data were normalized using edgeR 3.16.5. Heatmaps were generated using the R package pheatmap v.1.0.12, taking as input the normalized expression of ribosomal proteins and ribosome biogenesis factors (Table S9). List of ribosomal protein (RP) and ribosome biogenesis factors (RBF) were obtained from (Martinez-Seidel et al. 2020) and (Sáez-Vásquez and Delseny 2019) respectively. Gene expression was hierarchically clustered by row and column. All sequences were submitted to the Sequence Read Archive (SRA): SUB9740608.

Supplemental References:

Bouyssie D, Hesse AM, Mouton-Barbosa E, Rompais M, Macron C, Carapito C, Gonzalez de Peredo A, Coute Y, Dupierris V, Burel A et al. 2020. Proline: an efficient and user-friendly software suite for large-scale proteomics. *Bioinformatics (Oxford, England)* **36**: 3148-3155.

Martinez-Seidel F, Beine-Golovchuk O, Hsieh YC, Kopka J. 2020. Systematic Review of Plant Ribosome Heterogeneity and Specialization. *Front Plant Sci* **11**: 948.

Micol-Ponce R, Sarmiento-Manus R, Fontcuberta-Cervera S, Cabezas-Fuster A, de Bures A, Saez-Vasquez J, Ponce MR. 2020. SMALL ORGAN4 Is a Ribosome Biogenesis Factor Involved in 5.8S Ribosomal RNA Maturation. *Plant Physiol* **184**: 2022-2039.

Micol-Ponce R, Sarmiento-Manus R, Ruiz-Bayon A, Montacie C, Saez-Vasquez J, Ponce MR. 2018. Arabidopsis RIBOSOMAL RNA PROCESSING7 is required for 18S rRNA maturation. *Plant Cell* **30**: 2855–2872.

Sáez-Vásquez J, Delseny M. 2019. Ribosome Biogenesis in Plants: from Functional 45S Ribosomal DNA Organization to Ribosome Assembly Factors. *The Plant Cell* **31**: 1945–1967.

Wisniewski JR, Zougman A, Nagaraj N, Mann M. 2009. Universal sample preparation method for proteome analysis. *Nature methods* **6**: 359-362.
