# Supplementary figures and images for: Upon heat stress processing of ribosomal RNA precursors into mature rRNAs is compromised after cleavage at primary P site in Arabidopsis thaliana
Source: RNA Biol. 2022 May 6;19(1):719–34. doi: 10.1080/15476286.2022.2071517 (PMC9090299; doi:10.1080/15476286.2022.2071517)

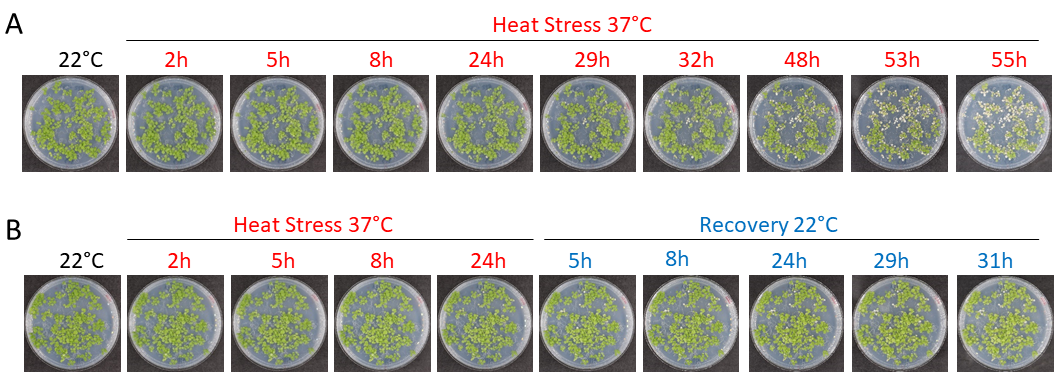

Supplement: Supplemental Material [file KRNB_A_2071517_SM7003.zip › Figure S1.tif]

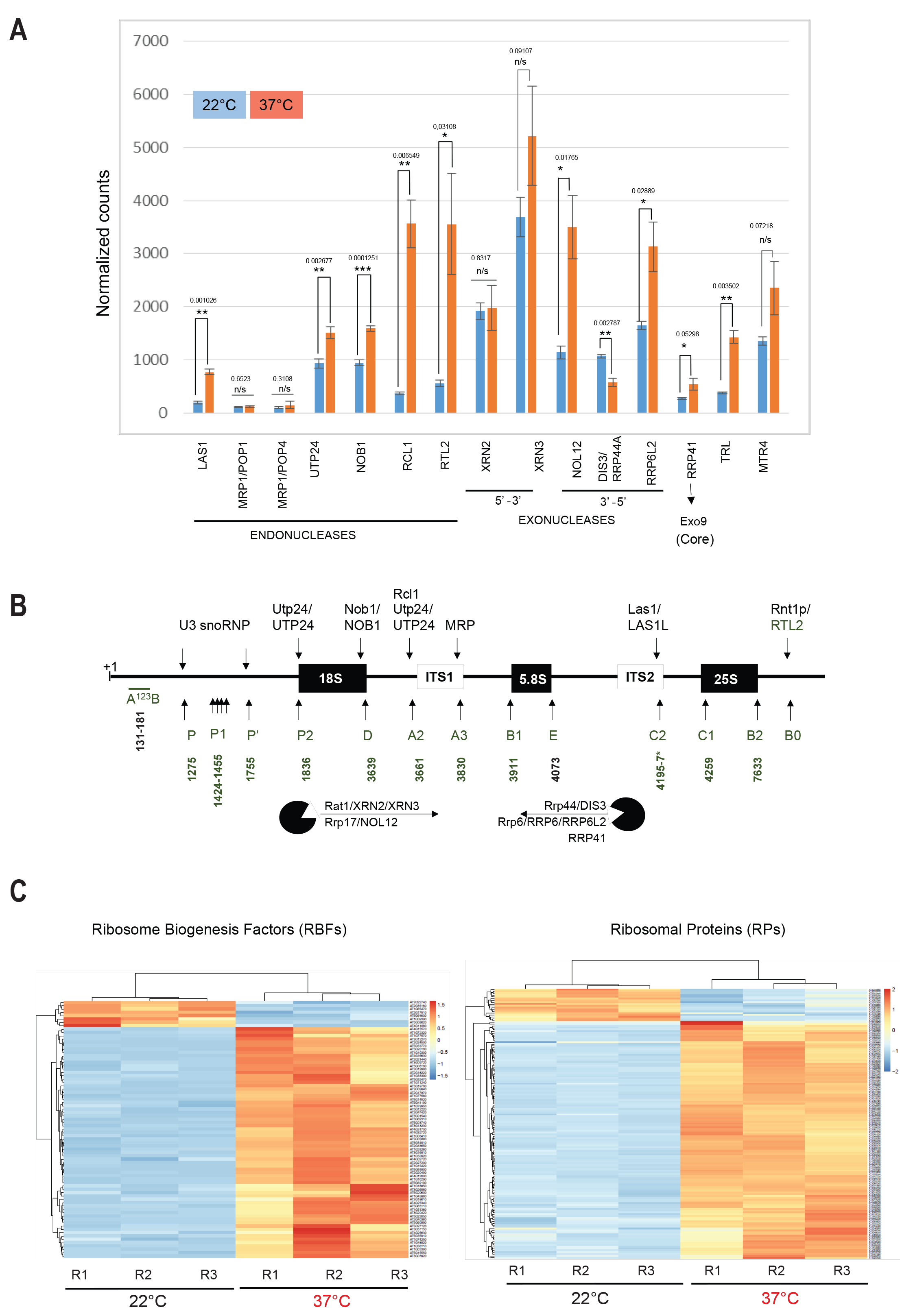

Supplement: Supplemental Material [file KRNB_A_2071517_SM7003.zip › Figure S10.tif]

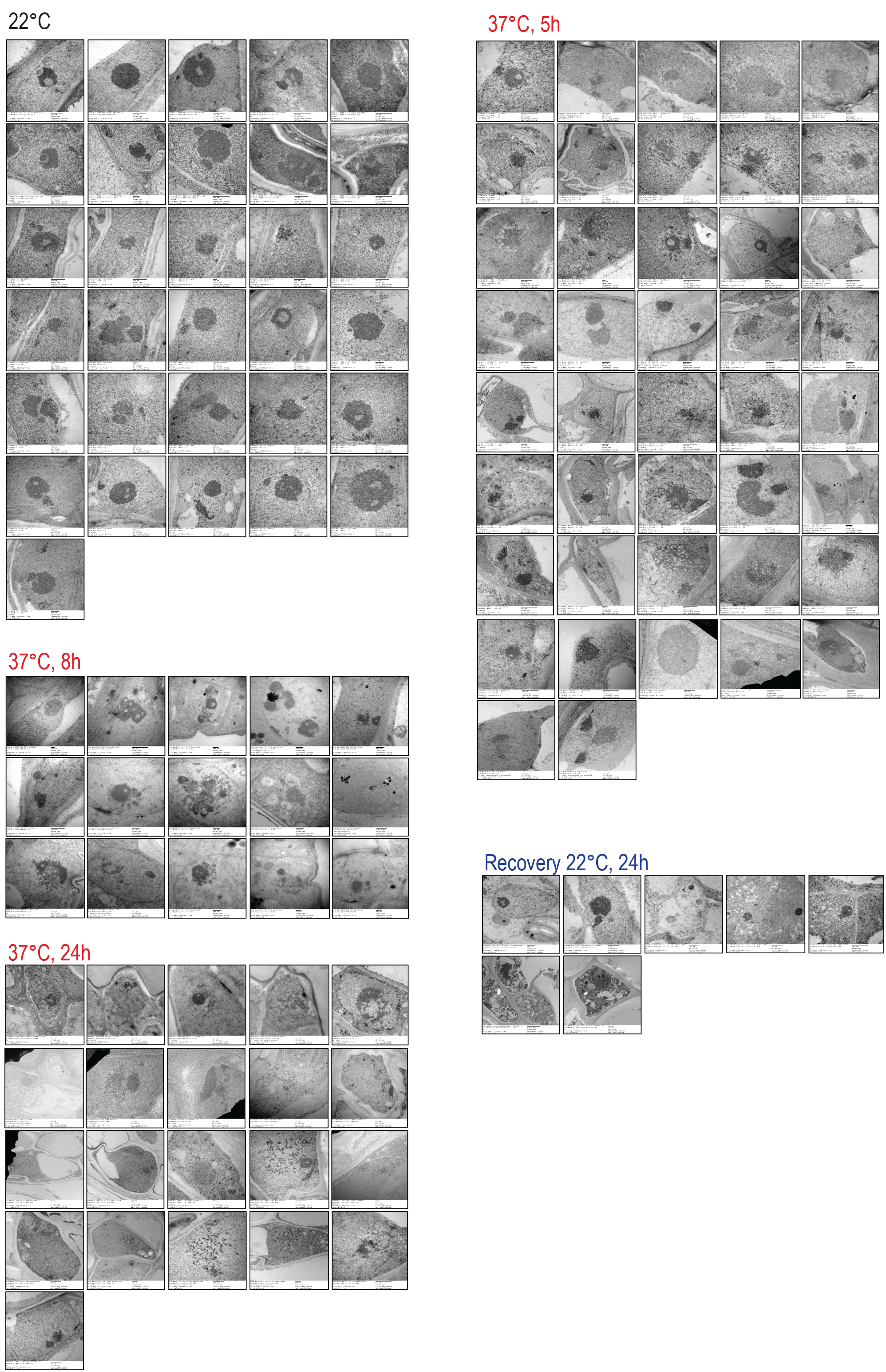

Supplement: Supplemental Material [file KRNB_A_2071517_SM7003.zip › Figure S2.tif]

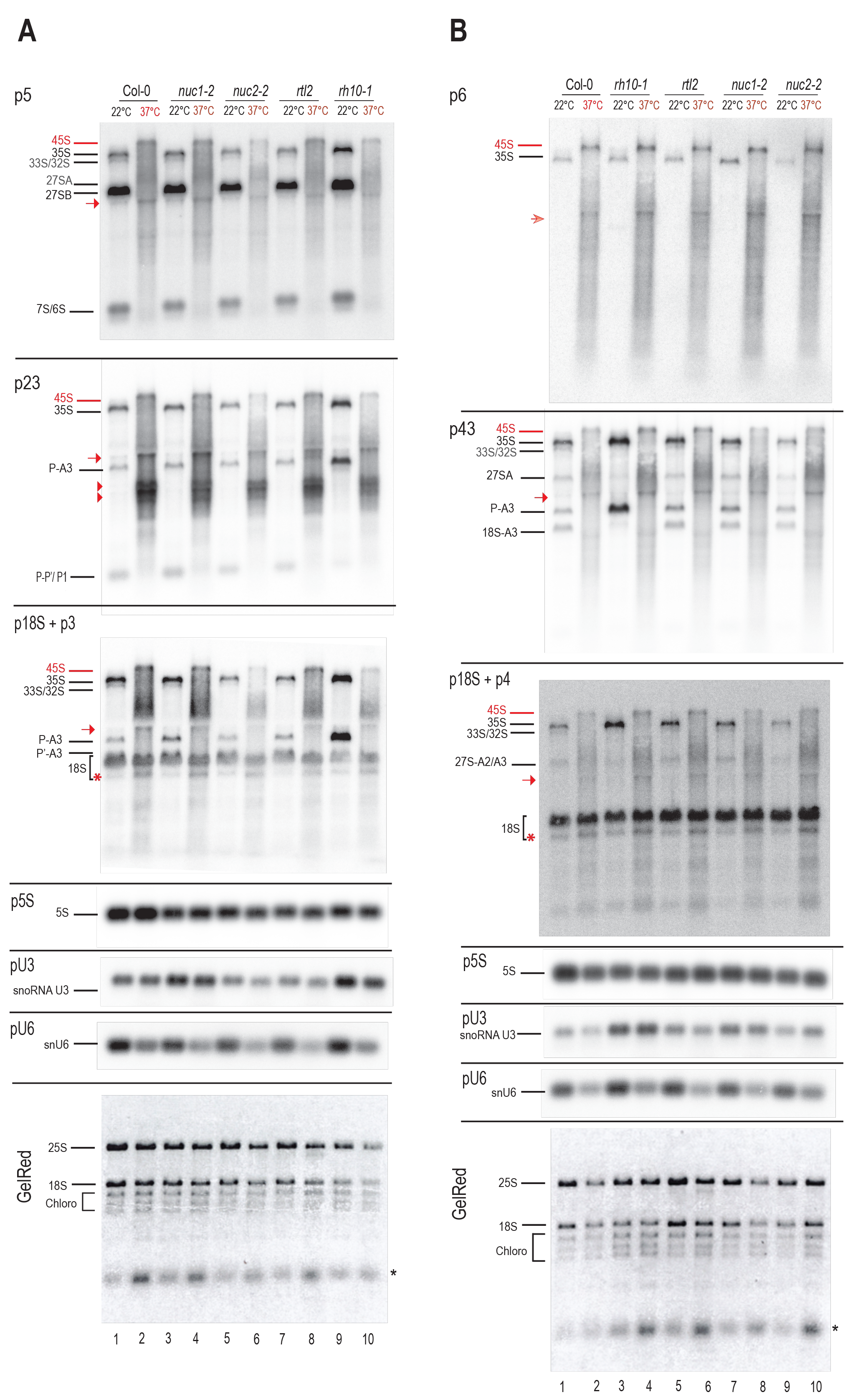

Supplement: Supplemental Material [file KRNB_A_2071517_SM7003.zip › Figure S3.tif]

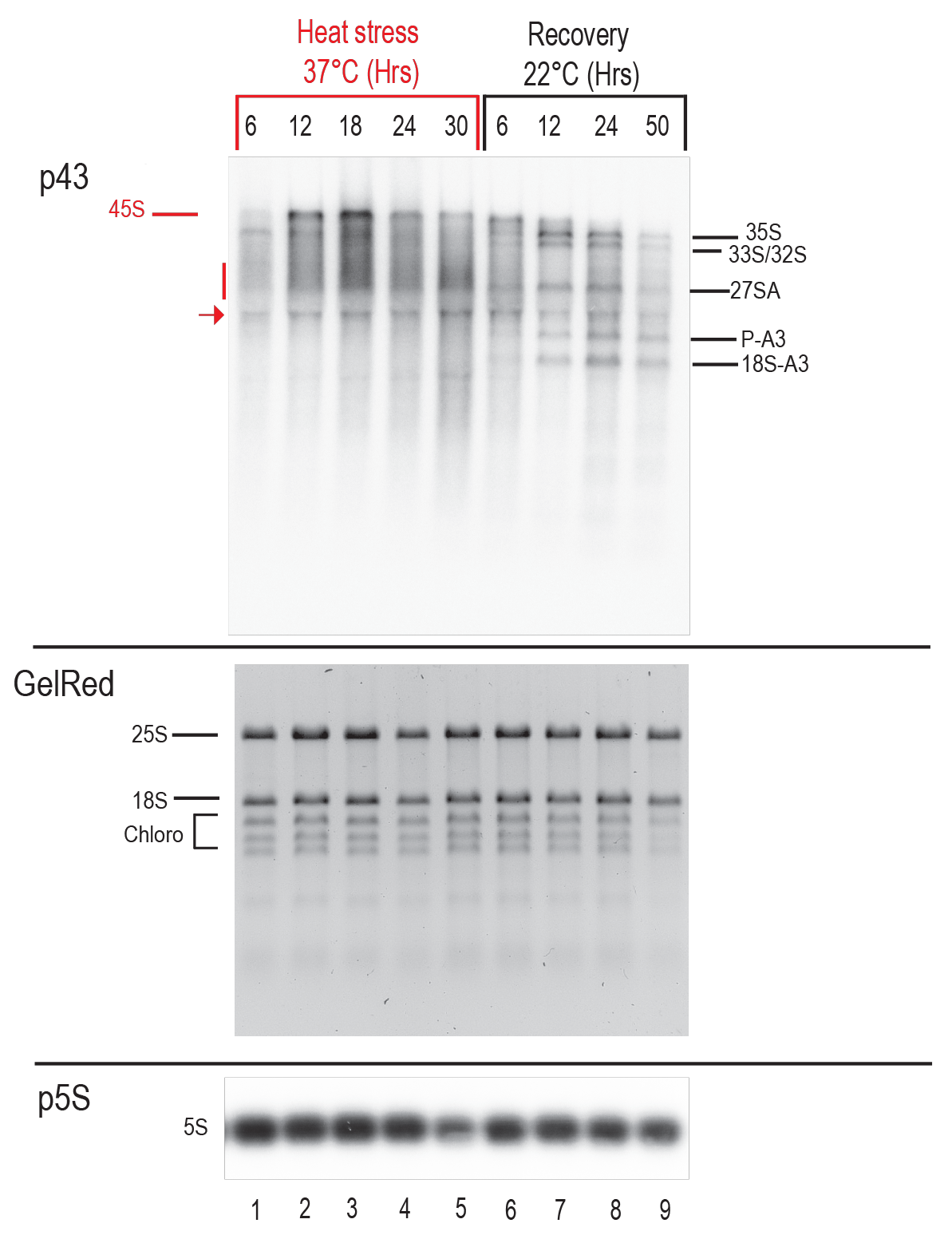

Supplement: Supplemental Material [file KRNB_A_2071517_SM7003.zip › Figure S4.tif]

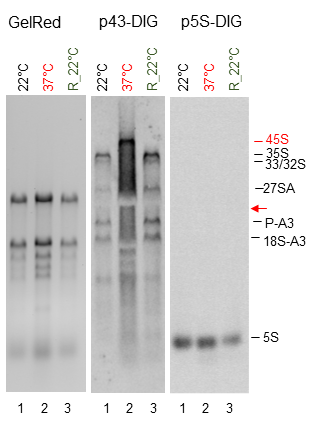

Supplement: Supplemental Material [file KRNB_A_2071517_SM7003.zip › Figure S5.tif]

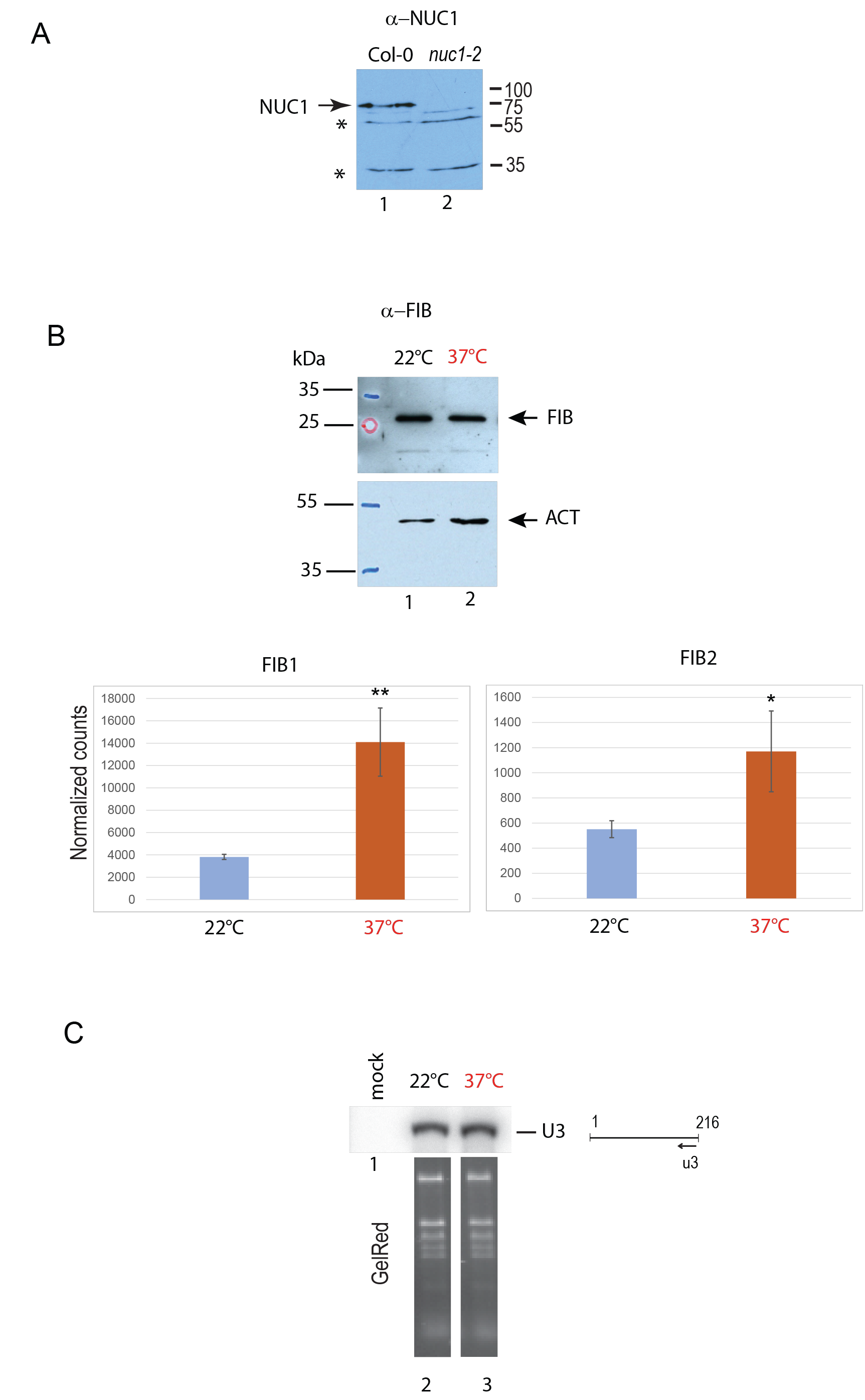

Supplement: Supplemental Material [file KRNB_A_2071517_SM7003.zip › Figure S6.tif]

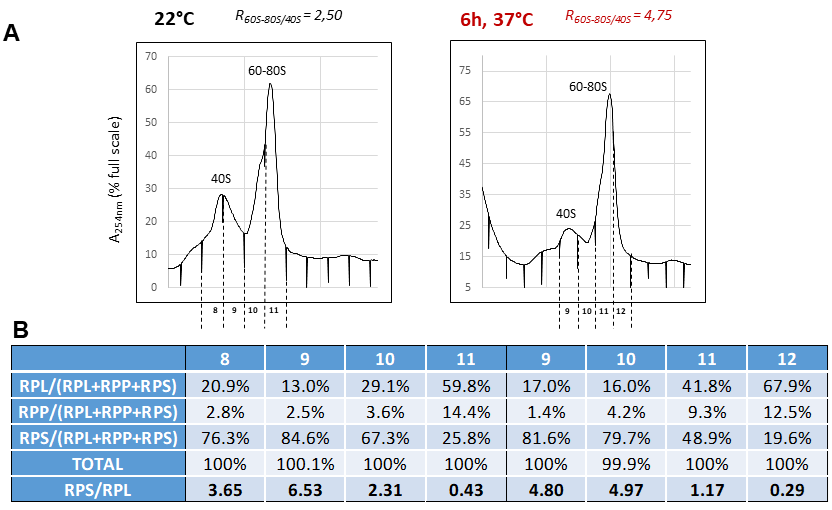

Supplement: Supplemental Material [file KRNB_A_2071517_SM7003.zip › Figure S7.tif]

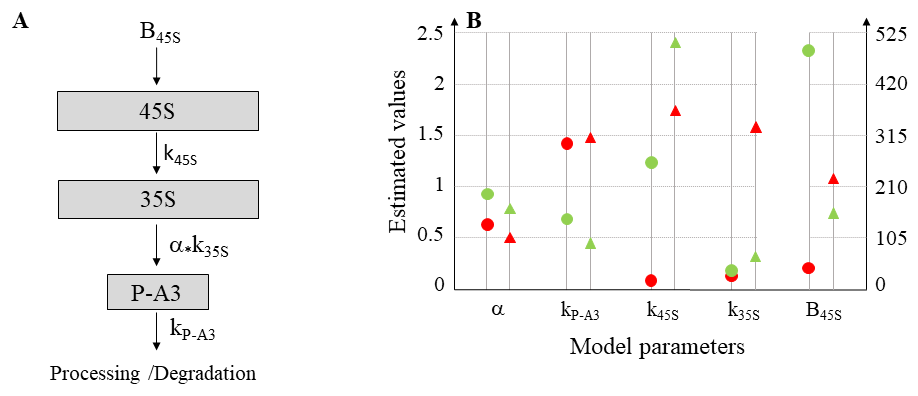

Supplement: Supplemental Material [file KRNB_A_2071517_SM7003.zip › Figure S8.tif]

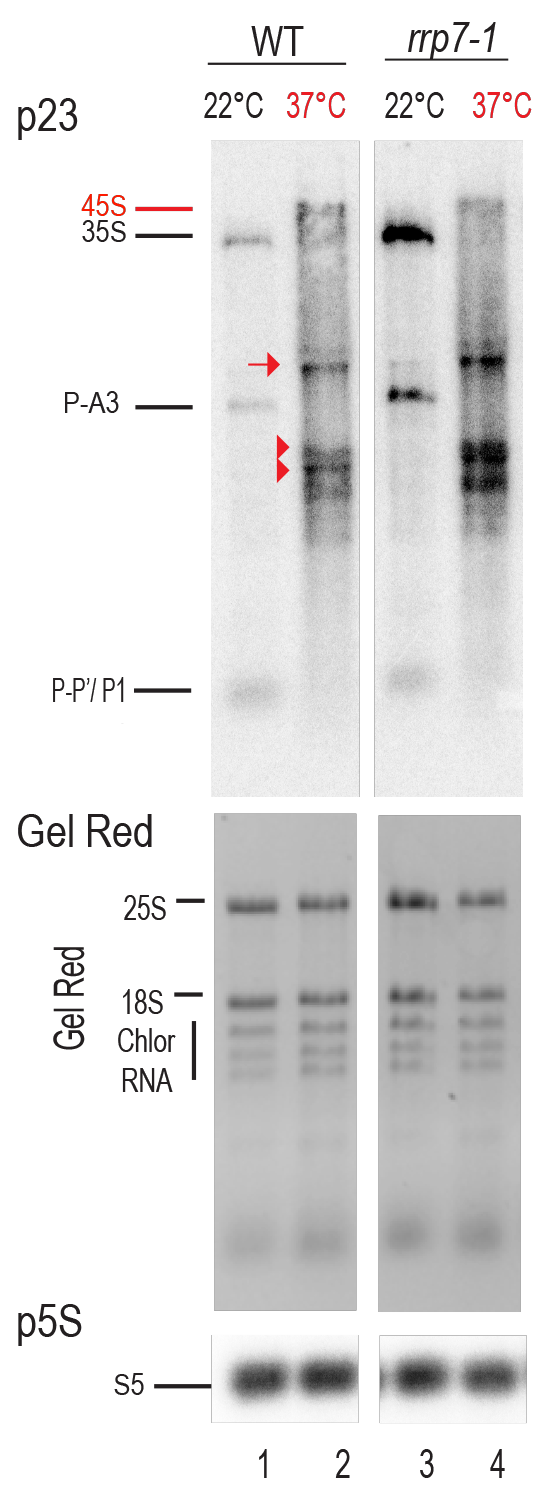

Supplement: Supplemental Material [file KRNB_A_2071517_SM7003.zip › Figure S9.tif]
